# Erratum: Symmetry-based indicators of band topology in the 230 space groups

**DOI:** 10.1038/s41467-017-00724-z

**Published:** 2017-10-10

**Authors:** Hoi Chun Po, Ashvin Vishwanath, Haruki Watanabe

**Affiliations:** 1 Department of Physics, University of California, Berkeley, CA 94720 USA; 2 Department of Physics, Harvard University, Cambridge, MA 02138 USA; 3 Department of Applied Physics, University of Tokyo, Tokyo, 113-8656 Japan

*Nature Communications* **8**:50 doi: 10.1038/s41467-017-00133-2; Article published online: 30 Jun 2017

Due to a production error, this Article was originally published with an incorrect title. The title should have been “Symmetry-based indicators of band topology in the 230 space groups”, not “Complete theory of symmetry-based indicators of band topology”. This has now been corrected in both the PDF and HTML versions of the Article.

