# Supplementary material for: Symmetry-based indicators of band topology in the 230 space groups
Source: Nat Commun. 2017 Jun 30;8:50. doi: 10.1038/s41467-017-00133-2 (PMC5493703; doi:10.1038/s41467-017-00133-2)
Supplement: Supplementary file 1 — Supplementary Information [file 41467_2017_133_MOESM1_ESM.pdf]

Title of file for HTML: Supplementary Information
Description: Supplementary Figure, Supplementary Tables, Supplementary Notes and Supplementary References

Title of file for HTML: Peer Review File
Description:

Supplementary Note 1. Review of Symmetries in Band Structures

Here, we briefly review some notions and results concerning the consequences of symmetries on band structure. The discussion here will closely mirror that of the 1D example given in the main text, but we will assume a general 3D setting from the outset.

Little group and its representation

Consider a system of noninteracting fermions symmetric under a purely spatial symmetry group $\mathcal{G}$ with the lattice translation subgroup T. An element $g \in \mathcal{G}$ that maps a point $\mathbf{x} \in \mathbb{R}^3$ to $g(\mathbf{x}) = p_g\mathbf{x} + \mathbf{t}_g \in \mathbb{R}^3$ may be characterized by an orthogonal matrix p_g and a vector $\mathbf{t}_g$. Thanks to the lattice translation T, it is natural to label the eigenenergy $E_m(\mathbf{k})$ by the band index m and the wavevector $\mathbf{k} \in$ BZ. The notion of energy bands, however, becomes ambiguous whenever they cross, since the underlying wave function of a single band will generally become discontinuous. Instead, it is more natural to consider a set of entangled bands, separated from all others by band gaps above and below, as a single entity.

Whether a given set of energy bands can be isolated from the others dictate whether or not the system can be insulating, and therefore is a fundamental question in the study of electronic band structures. High-symmetry momenta play a crucial role in this analysis. The subgroup of $\mathcal{G}$ that leaves a momentum $\mathbf{k} \in$ BZ invariant (up to a reciprocal lattice vector $\mathbf{G}$) is known as the little group of $\mathbf{k}$, which is commonly denoted by $\mathcal{G}_{\mathbf{k}}$ [1]. As a set, we have

$$\mathcal{G}_{\mathbf{k}} = \{g \in \mathcal{G} \ : \ p_g\mathbf{k} = \mathbf{k} + {}^{\exists}\mathbf{G}\}. \tag{1}$$

By definition, lattice translations T automatically form a subgroup of $\mathcal{G}_{\mathbf{k}}$, and we say $\mathbf{k}$ is a high-symmetry momentum whenever $\mathcal{G}_{\mathbf{k}}$ contains any element aside from lattice translations. Note that when we say 'all high-symmetry momenta', we include all high-symmetry points, lines and planes in the BZ.

Generally, a spatial symmetry g demands $E_m(p_g\mathbf{k}) = E_m(\mathbf{k})$, and the corresponding wave functions are similarly related. When $g \in \mathcal{G}_{\mathbf{k}}$, the constraint becomes a local condition in momentum space. This implies the wave functions furnish a representation of $\mathcal{G}_{\mathbf{k}}$ [1]. Such representations encode the transformation properties of the BS at $\mathbf{k}$, and can be generally decomposed into a direct sum of irreducible representations (irreps) $u_{\mathbf{k}}^{\alpha}$, which have been exhaustively tabulated in Supplementary Ref. [1] for both spinless and spinful fermions. Insofar as symmetry properties are concerned, we can label a BS at $\mathbf{k}$ by the set of non-negative integers

$$\{n_{\mathbf{k}}^{\alpha} \ : \ \alpha = 1, \dots, D_{\mathbf{k}}\}, \tag{2}$$

where $n_{\mathbf{k}}^{\alpha}$ denotes the number of times the α-th irrep appears in the BS, and $D_{\mathbf{k}}$ denotes the number of irreps. Note that such symmetry labeling makes no reference to the detailed energetics of the system, i.e. the labels are insensitive to the energetic arrangement of the individual bands within the BS.

Types of momenta and compatibility relations

A priori, a BS will carry independent symmetry labels $\{n_{\mathbf{k}}^{\alpha}\}$ and $\{n_{\mathbf{k}'}^{\beta}\}$ at distinct high-symmetry momenta $\mathbf{k}$ and $\mathbf{k}'$. However, symmetries and continuity can cast extra constraints on such assignment [1]. To see this concretely, let us classify all points in BZ into a finite number of 'types': we say $\mathbf{k}_1$ and $\mathbf{k}_2$ are of the same type if either $\mathbf{k}_1$ and $\mathbf{k}_2$ are symmetry-related, i.e., $\mathbf{k}_2 = p_{g_0}\mathbf{k}_1 + {}^{\exists}\mathbf{G}$, or each point of the line connecting $\mathbf{k}_1$ and $\mathbf{k}_2$ has the same $\mathcal{G}_{\mathbf{k}}$ as $\mathcal{G}_{\mathbf{k}_1} = \mathcal{G}_{\mathbf{k}_2}$. For example, imagine a line invariant under a n-fold rotation (or screw) C_n. Any point on this line (except for possibly the end points) has the identical little group generated by C_n and hence is of the same type. Similar situation arises for a plane invariant under a mirror (or a glide). By assumption, the existence of band gaps imply the representation content of a BS is invariant within such high-symmetry lines or planes, and therefore it suffices to specify the representations at one arbitrarily chosen $\mathbf{k}$ point for each type of momenta in the BZ. Therefore, a full set of symmetry labels of a BS can be obtained by specifying only those arising from a finite number of representatives. In addition, the BS is also labeled by the total number of bands in the BS, which we will denote by ν. Note that physically ν is simply the electron filling of the system when BS is interpreted as the set of filled bands in the full system. Aggregating all the labels $\{n_{\mathbf{k}}^{\alpha}\}$ and ν into a single quantity, we denote the representation content of a BS by $\mathbf{n}$, a set of D non-negative integers where $D = 1 + \sum_{\mathbf{k}} D_{\mathbf{k}}$. Here, we have chosen one arbitrary $\mathbf{k}$ for each type of the $\mathbf{k}$ vectors in the BZ.

Next, we note that a general $\mathbf{n}$ may not be compatible with the continuity of the bands and the gap condition we imposed on BS. To find the set of admissible labels $\{\mathbf{n}\}$, we introduce the notion of compatibility relations. [1] For any pair of infinitesimally close momenta $\mathbf{k}$ and $\mathbf{k} + \delta\mathbf{k}$, their little groups satisfy $\mathcal{G}_{\mathbf{k}+\delta\mathbf{k}} \leq \mathcal{G}_{\mathbf{k}}$ (assuming a proper choice of labeling for the two momenta). Since the irreps at the higher-symmetry momentum $\mathbf{k}$ can be decomposed into those of the lower-symmetry ones at $\mathbf{k} + \delta\mathbf{k}$, the symmetry labels $\{n_{\mathbf{k}+\delta\mathbf{k}}^{\alpha}\}$ are fully constrained by $\{n_{\mathbf{k}}^{\beta}\}$. This is captured by the compatibility relations, one for each value of α,

$$n_{\mathbf{k}+\delta\mathbf{k}}^{\alpha} = \sum_{\beta} c_{\alpha\beta}^{\mathbf{k},\delta\mathbf{k}} n_{\mathbf{k}}^{\beta}, \tag{3}$$

where the coefficients $c_{\alpha\beta}^{\mathbf{k},\delta\mathbf{k}}$ are non-negative integers. There is also a similar compatibility on the filling: $\nu =$

$\sum_\alpha \dim[u_{\mathbf{k}}^\alpha]\, n_{\mathbf{k}}^\alpha$, where $\dim[u_{\mathbf{k}}^\alpha]$ denotes the dimension of the irrep α (i.e. the number of bands involved).

As our target is to study BSs as global entities, it is instructive to collect all compatibility relations into a system of linear equations:

$$\mathcal{C}\mathbf{n} = 0, \tag{4}$$

where $\mathcal{C}$ is an integer-valued matrix with coefficients determined by those in Supplementary Eq. (3). By definition, any BS can be identified with an $\mathbf{n}$ satisfying Supplementary Eq. (4). Conversely, any set of D non-negative integers $\mathbf{n} \in \mathbb{Z}^D_{\geq 0}$ satisfying Supplementary Eq. (4) can be identified with a physical band structure (Supplementary Note 3).

Construction of irreps of $\mathcal{G}$

Given an irrep $u_{\mathbf{k}}^\alpha$ of $\mathcal{G}_{\mathbf{k}}$, one can construct an irrep of $\mathcal{G}$, which s called the induced representation. It is known that every irrep of $\mathcal{G}$ can be constructed in this way [1]. Here we review the construction in detail for the case where $u_{\mathbf{k}}^\alpha$ is a projective representation of $\mathcal{G}_{\mathbf{k}}$ due to the spin degrees of freedom.

Let $\{|\phi_{i,\mathbf{k}}^r\rangle\}_{i=1}^{\dim[u_{\mathbf{k}}^\alpha]}$ be the basis of an irrep $u_{\mathbf{x}}^r$ of $\mathcal{G}_{\mathbf{x}}$. Namely, $|\phi_{i,\mathbf{k}}^r\rangle$ transform under $h, h' \in \mathcal{G}_{\mathbf{k}}$ as

$$\hat{h}|\phi_{i,\mathbf{k}}^\alpha\rangle = \sum_j |\phi_{j,\mathbf{k}}^\alpha\rangle [u_{\mathbf{k}}^\alpha(h)]_{ji}, \tag{5}$$

$$u_{\mathbf{k}}^\alpha(h) u_{\mathbf{k}}^\alpha(h') = z_{h,h'} u_{\mathbf{k}}^\alpha(hh'), \tag{6}$$

$$\hat{h}(\hat{h}'|\phi_{i,\mathbf{k}}^\alpha\rangle) = z_{h,h'}(\hat{hh'})|\phi_{i,\mathbf{k}}^\alpha\rangle, \tag{7}$$

where $z_{g,g'} = \pm 1$ is the projective phase originating from the spin of fermions. For the spinless case $z_{g,g'}$ should be set to be 1.

Since elements $g \notin \mathcal{G}_{\mathbf{k}}$ change $\mathbf{k}$ to an inequivalent momentum $p_g\mathbf{k}$ by definition, $\hat{g}|\phi_{i,\mathbf{k}}^r\rangle$ cannot, in general, be expanded by $\{|\phi_{i,\mathbf{k}}^r\rangle\}_i$. The symmetry orbit of $\mathbf{k}$, $\{\mathbf{k}_\sigma\}_{\sigma=1}^{|\mathcal{G}/\mathcal{G}_{\mathbf{k}}|} = \{p_g\mathbf{k}\ :\ g \in \mathcal{G}\}$, is called the star of $\mathbf{k}$. We arbitrarily choose a complete set of representatives $\{g_\sigma\}_{\sigma=1}^{|\mathcal{G}/\mathcal{G}_{\mathbf{k}}|}$ ($g_{\sigma=1} = e$) of $\mathcal{G}/\mathcal{G}_{\mathbf{k}}$ that satisfies $\mathbf{k}_\sigma \equiv p_{g_\sigma}\mathbf{k}$ and define $|\phi_{i,\mathbf{k}_\sigma}^\alpha\rangle \equiv \hat{g}_\sigma|\phi_{i,\mathbf{k}}^\alpha\rangle$. The set $\{|\phi_{i,\mathbf{k}_\sigma}^r\rangle\}_{i,\sigma}$ serves as the basis of the representation of $\mathcal{G}$. To see how $|\phi_{i,\mathbf{k}_\sigma}^\alpha\rangle$ transforms under a general element $g \in \mathcal{G}$, note that $gg_\sigma \in \mathcal{G}$ can be uniquely decomposed into a product of $g_{\sigma'} \in \mathcal{G}/\mathcal{G}_{\mathbf{k}}$ and $h \in \mathcal{G}_{\mathbf{k}}$. Therefore, step-by-step, we have

$$\begin{aligned}
\hat{g}|\phi_{i,\mathbf{k}_\sigma}^\alpha\rangle &= \hat{g}(\hat{g}_\sigma|\phi_{i,\mathbf{k}}^\alpha\rangle) \\
&= z_{g,g_\sigma}(\hat{gg_\sigma})|\phi_{i,\mathbf{k}}^\alpha\rangle = z_{g,g_\sigma}(\hat{g_{\sigma'}h})|\phi_{i,\mathbf{k}}^\alpha\rangle \\
&= \frac{z_{g,g_\sigma}}{z_{g_{\sigma'},h}} \sum_{i'} \hat{g}_{\sigma'}|\phi_{i',\mathbf{k}}^\alpha\rangle [u_{\mathbf{k}}^\alpha(h)]_{i'i} \\
&= \frac{z_{g,g_\sigma}}{z_{g_{\sigma'},h}} \sum_{i'} \hat{g}_{\sigma'}|\phi_{i',\mathbf{k}}^\alpha\rangle [u_{\mathbf{k}}^\alpha(h)]_{i'i} \\
&= \sum_{\sigma',i'} |\phi_{i',\mathbf{k}_{\sigma'}}^\alpha\rangle [U^\alpha(g)]_{\sigma'i',\sigma i},
\end{aligned} \tag{8}$$

where

$$[U^\alpha(g)]_{\sigma'j,\sigma i} = \delta''_{\mathbf{k}_{\sigma'},p_g\mathbf{k}_\sigma} \frac{z_{g,g_\sigma}}{z_{g_{\sigma'},g_{\sigma'}^{-1}gg_\sigma}} [u_{\mathbf{k}}^\alpha(g_{\sigma'}^{-1}gg_\sigma)]_{ji} \tag{9}$$

and $\delta''_{\mathbf{k}_1,\mathbf{k}_2}$ is 1 only when $\mathbf{k}_1 = \mathbf{k}_2$ modulo a reciprocal lattice vector. This is the induced representation of $\mathcal{G}$ constructed from $u_{\mathbf{k}}^\alpha$ of $\mathcal{G}_{\mathbf{k}}$. It has a nice property that U^α is irreducible whenever $u_{\mathbf{k}}^\alpha$ is.

Time-reversal symmetry

Here we will follow the discussion in Supplementary Ref. [1] on the consequence of the TR symmetry $\mathcal{T}$ commuting with every element of G. We will write $\hat{\mathcal{T}}^2 = (\eta_{\mathcal{T}})^{\hat{N}}$ where $\hat{N}$ is the number of fermions and $\eta_{\mathcal{T}} = -1$ ($\eta_{\mathcal{T}} = +1$) for the spinful (spinless) case.

1. *General case*

Let us start with a finite group G in general. Suppose that $\{|i\rangle\}_{i=1}^d$ ($d \equiv \dim[u]$) is a basis of an irrep u of G:

$$\hat{g}|i\rangle = |j\rangle u_{ji}(g). \tag{10}$$

Then $\{\hat{\mathcal{T}}|i\rangle\}_{i=1}^d$ is a basis of the conjugate representation $u^*(g)$:

$$\hat{g}(\hat{\mathcal{T}}|i\rangle) = (\hat{\mathcal{T}}|j\rangle) u_{ji}^*(g). \tag{11}$$

When u and u^* are different irreps, the group $G + \mathcal{T}G$ is simply represented by

$$D(g) = \begin{pmatrix} u & 0 \\ 0 & u^* \end{pmatrix}, \quad D(T) = \begin{pmatrix} 0 & \eta_{\mathcal{T}} \\ 1 & 0 \end{pmatrix}. \tag{12}$$

The situation is different when u and u^* are the same irrep, i.e, $u^*(g) = v^\dagger u(g) v$ for a unitary matrix v. Using the definition of v twice, we have $(vv^*)u(g) = u(g)(vv^*)$ for every g. Since u is irreducible, we see $vv^* = \xi\eta_{\mathcal{T}}\mathbb{1}_d$ for $\xi = \pm 1$. In other words,

$$v^T = (v^*)^\dagger = \xi\eta_{\mathcal{T}} v, \quad \xi, \eta_{\mathcal{T}} = \pm 1. \tag{13}$$

It is easy to see that the combination $|\bar{i}\rangle \equiv (\hat{\mathcal{T}}|j\rangle) v_{ji}^\dagger$ transforms in the same way as $|i\rangle$ under G:

$$\hat{g}|\bar{i}\rangle = |\bar{j}\rangle u_{ji}(g). \tag{14}$$

Hence the question is if $|i\rangle$ and $|\bar{i}\rangle$ are the same. To see this, let us evaluate the inner-product $G_{ij} \equiv (|i\rangle, |\bar{j}\rangle)$. Since $\hat{g}$ is unitary, we have

$$G_{ij} = (|i\rangle, |\bar{j}\rangle) = (\hat{g}|i\rangle, \hat{g}|\bar{j}\rangle) = [u(g)^\dagger G u(g)]_{ij}, \tag{15}$$

i.e., G and $u(g)$ commute for every g and we must have $G = c\,\mathbb{1}_d$. To compute c, note that

$$\hat{\mathcal{T}}|i\rangle = |\bar{j}\rangle v_{ji}, \tag{16}$$

$$\hat{\mathcal{T}}|\bar{i}\rangle = \eta_{\mathcal{T}}|j\rangle (v^T)_{ji} = \xi|j\rangle v_{ji}. \tag{17}$$

The second line follows from the first by applying $\hat{\mathcal{T}}$ and using Supplementary Eq. (13). Hence

$$c = \frac{1}{d}\sum_{i=1}^{d}(|i\rangle, |\bar{i}\rangle) = \frac{1}{d}\sum_{i=1}^{d}(\hat{\mathcal{T}}|\bar{i}\rangle, \hat{\mathcal{T}}|i\rangle) \\ = \frac{\xi}{d}(vv^\dagger)_{j,j'}\,(|j'\rangle, |\bar{j}\rangle) = \xi c. \tag{18}$$

Namely, when $\xi = -1$, c vanishes and $\{|i\rangle\}_{i=1}^{d}$ and $\{\hat{\mathcal{T}}|i\rangle\}_{i=1}^{d}$ are orthogonal. On the other hand, when $\xi = 1$, c is finite and $\{|i\rangle\}_{i=1}^{d}$ and $\{\hat{\mathcal{T}}|i\rangle\}_{i=1}^{d}$ are the same state.

To compute ξ from the character $\chi(g) = \mathrm{tr}[u(g)]$, we use the following identity

$$z_{g,g}u(g^2) = u(g)u(g) = [u^*(g)]^*u(g) \\ = [v^\dagger u(g)v]^*u(g) = \xi\eta_{\mathcal{T}}vu^*(g)v^*u(g). \tag{19}$$

We used Supplementary Eq. (13) in the last line. Therefore,

$$\eta_{\mathcal{T}}\frac{1}{|G|}\sum_g z_{g,g}\chi(g^2) = \xi\frac{1}{|G|}\sum_g v_{ij}u^*_{jk}(g)v^*_{kl}u_{li}(g) \\ = \xi\frac{1}{d}(vv^\dagger)_{i,i} = \xi. \tag{20}$$

where, in the second last line we used the orthogonality of irreps $\sum_g u^{(\alpha)}_{ij}(g)u^{(\beta)}_{kl}(g)^* = (|G|/d)\delta_{ik}\delta_{jl}\delta^{\alpha\beta}$. From this orthogonality, it is also clear that $\sum_g z_{g,g}\chi(g^2) = 0$ when u and u^* are different irreps.

To summarize, to see if the TR symmetry changes of degeneracy for the irrep u of G, one should compute

$$\eta_{\mathcal{T}}\frac{1}{|G|}\sum_{g\in G} z_{g,g}\chi(g^2) \\ = \begin{cases} +1 & : \text{Degeneracy is unchanged.} \\ -1 & : \text{Two } u\text{'s are paired under TR.} \\ 0 & : u \text{ and } u^* \text{ are different and are paired.}\end{cases} \tag{21}$$

This is called Wigner's test in the literature [1].

2. TR pairing of u_k^α

Let us apply this result to irreps $u^\alpha_{\mathbf{k}}$ of $\mathcal{G}_{\mathbf{k}}$. If there is no element $g \in \mathcal{G}$ such that $p_g\mathbf{k} = -\mathbf{k}$ modulo a reciprocal lattice vector $\mathbf{G}$, then the TR symmetry just implies an additional degeneracy between $u^\alpha_{\mathbf{k}}$ and $u^\alpha_{-\mathbf{k}}$ and this case is easily handled. Thus, suppose that there is at least one $g \in \mathcal{G}$ such that $p_g\mathbf{k} = -\mathbf{k} + {}^{\exists}\mathbf{G}$.

As explained earlier, an irrep $u^\alpha_{\mathbf{k}}$ of $\mathcal{G}_{\mathbf{k}}$ induced an irrep U^α of $\mathbf{G}$, see Supplementary Eq. (9). Its character can be expressed as

$$\mathrm{tr}[U^\alpha(g)] = \sum_\sigma \delta''_{\mathbf{k}_\sigma, p_g\mathbf{k}_\sigma}\frac{z_{g,g_\sigma}}{z_{g_\sigma, g_\sigma^{-1}gg_\sigma}}\mathrm{tr}[u^\alpha_{\mathbf{k}}(g_\sigma^{-1}gg_\sigma)] \\ = \sum_\sigma\sum_{h\in\mathcal{G}_{\mathbf{k}}}\delta_{g,g_\sigma hg_\sigma^{-1}}\frac{z_{g,g_\sigma}}{z_{g_\sigma,h}}\mathrm{tr}[u^\alpha_{\mathbf{k}}(h)] \\ = \sum_\sigma\sum_{h\in\mathcal{G}_{\mathbf{k}_\sigma}}\delta_{g,h}\mathrm{tr}[u^\alpha_{\mathbf{k}_\sigma}(h)] \\ = \sum_\sigma\sum_{h\in\mathcal{G}_{\mathbf{k}_\sigma}}\delta_{g,h}\chi^\alpha_{\mathbf{k}_\sigma}(h), \tag{22}$$

where $u^\alpha_{\mathbf{k}_\sigma}(h) \equiv \frac{z_{h,g_\sigma}}{z_{g_\sigma,g_\sigma^{-1}g_hg_\sigma}}u^\alpha_{\mathbf{k}}(g_\sigma^{-1}hg_\sigma)$ and $\chi^\alpha_{\mathbf{k}_\sigma}(h) = \mathrm{tr}[u^\alpha_{\mathbf{k}_\sigma}(h)]$. Hence,

$$\frac{1}{|\mathcal{G}|}\sum_{g\in\mathcal{G}} z_{g,g}\mathrm{tr}[U^\alpha(g^2)] \\ = \frac{1}{|\mathcal{G}|}\sum_{g\in\mathcal{G}}\sum_\sigma\sum_{h\in\mathcal{G}_{\mathbf{k}_\sigma}}\delta_{g^2,h}z_{g,g}\chi^\alpha_{\mathbf{k}_\sigma}(h) \\ = \frac{1}{|\mathcal{G}_{\mathbf{k}}|}\sum_{g\in\mathcal{G}} z_{g,g}\chi^\alpha_{\mathbf{k}}(g^2) \\ = \frac{1}{|\mathcal{G}_{\mathbf{k}}/T|}\sum_{g\in\mathcal{G}/T}\delta''_{p_g\mathbf{k},-\mathbf{k}}\,z_{g,g}\chi^\alpha_{\mathbf{k}}(g^2) \tag{23}$$

To go to the third line, we used the fact that contributions from each star of $\mathbf{k}$ are identical and replaced $\sum_\sigma$ by the factor $|\mathcal{G}|/|\mathcal{G}_{\mathbf{k}}|$. To go to the last line, we performed the sum over the translation subgroup T, which results in the constraints $\sum_{\mathbf{t_R}\in T} e^{-i\mathbf{k}\cdot(p_g\mathbf{t_R}+\mathbf{t_R})} = |T|\delta''_{p_g\mathbf{k},-\mathbf{k}}$. Therefore, the TR index can be computed given by [1]

$$\eta_{\mathcal{T}}\frac{1}{|\mathcal{G}_{\mathbf{k}}/T|}\sum_{g\in\mathcal{G}/T}\delta''_{p_g\mathbf{k},-\mathbf{k}}z_{g,g}\chi^\alpha_{\mathbf{k}}(g^2) \\ = \begin{cases} +1 & : \text{Degeneracy is unchanged.} \\ -1 & : \text{Two } u^\alpha_{\mathbf{k}}\text{'s are paired under TR.} \\ 0 & : u^\alpha_{\mathbf{k}} \text{ is paired with another irrep } u^\beta_{\mathbf{k}}.\end{cases} \tag{24}$$

As explained in the main text, when an irrep $u^\alpha_{\mathbf{k}}$ is paired with a different irrep $u^\beta_{\mathbf{k}}$ under TR, we simply add to $\mathcal{C}$ an additional compatibility relation $n^\alpha_{\mathbf{k}} = n^\beta_{\mathbf{k}}$; when $u^\alpha_{\mathbf{k}}$ is paired with itself, we demand $n^\alpha_{\mathbf{k}}$ to be an even integer, which can be achieved by redefining $\tilde{n}^\alpha_{\mathbf{k}} \equiv n^\alpha_{\mathbf{k}}/2$ and a corresponding rewriting of $\mathcal{C}$ in terms of $\tilde{\mathbf{n}}$.

Supplementary Note 2. Atomic Insulators as Band Structures

In Supplementary Note 1, we characterized each BS by $\mathbf{n}$, the set of integers $n_{\mathbf{k}}^{\alpha}$ that specifies the representation contents of the BS. In this section, we derive a general formula that gives $\mathbf{n}$ for each AI.

Wyckoff position and site symmetry representations

An AI is specified by the location of the sites on which atomic orbitals sit, and the type of orbitals on each site. Mathematically, these two inputs correspond to the choice of a Wyckoff position and the representation of the site symmetry group of a site in the Wyckoff position.

Just as we defined the little group of $\mathbf{k}$, let us define the little group of $\mathbf{x}$ as the subgroup of $\mathcal{G}$ that leaves $\mathbf{x}$ invariant. We call it the site symmetry group $\mathcal{G}_{\mathbf{x}}$ of $\mathbf{x}$. As a set, we have

$$\mathcal{G}_{\mathbf{x}} = \{h \in \mathcal{G} \mid h(\mathbf{x}) \equiv p_h \mathbf{x} + \mathbf{t}_h = \mathbf{x}\}. \tag{25}$$

Points in real space are classified based on their little group. Namely, two points $\mathbf{x}_1$ and $\mathbf{x}_2$ belong to the same Wyckoff position iff there exists $g \in \mathcal{G}$ such that $\mathcal{G}_{\mathbf{x}_2} = g\mathcal{G}_{\mathbf{x}_1}g^{-1}$. The full list of different Wyckoff positions (in the real space) is available in Supplementary Ref. [2].

Let us pick a site $\mathbf{x}$ in the unit cell UC. By definition, elements of $\mathcal{G}$ not belonging to $\mathcal{G}_{\mathbf{x}}$ will move $\mathbf{x}$. The crystallographic orbit $\{g(\mathbf{x})|g \in \mathcal{G}\}$ defines a $\mathcal{G}$-symmetric lattice $L_{\mathbf{x}}$. Let $\{\mathbf{x}_\sigma\}_{\sigma=1,2,\ldots}$ ($\mathbf{x}_1 \equiv \mathbf{x}$) be the lattice points in UC. We choose $\{g_\sigma\}_{\sigma=1,2,\ldots}$ from $\mathcal{G}$ in such a way that $g_{\sigma=1} = e$ ($e \in \mathcal{G}$ is the identity) and $g_\sigma(\mathbf{x}) = \mathbf{x}_\sigma$ for $\sigma = 2, 3, \ldots$. Namely, $\{g_\sigma\}_{\sigma=1,2,\ldots}$ is a complete set of representatives of $\mathcal{W}_{\mathbf{x}} \equiv (\mathcal{G}/\mathcal{G}_{\mathbf{x}})/T$.

We want to introduce an orbital on every site of $L_{\mathbf{x}}$ in a symmetric manner. To that end, let us first put states $\{|\phi_{\mathbf{x},i,\mathbf{k}}^{r}\rangle\}_{i=1}^{\dim[u_{\mathbf{x}}^{r}]}$ on $\mathbf{x}$ that obey an irrep $u_{\mathbf{x}}^{r}$ of $\mathcal{G}_{\mathbf{x}}$:

$$\hat{h}|\phi_{\mathbf{x},i,\mathbf{k}}^{r}\rangle = \sum_j |\phi_{\mathbf{x},j,p_h\mathbf{k}}^{r}\rangle [u_{\mathbf{x}}^{r}(h)]_{ji}, \tag{26}$$

$$u_{\mathbf{x}}^{r}(h)u_{\mathbf{x}}^{r}(h') = z_{h,h'}u_{\mathbf{x}}^{r}(hh'), \tag{27}$$

$$\hat{h}(\hat{h}'|\phi_{\mathbf{x},i,\mathbf{k}}^{r}\rangle) = z_{h,h'}(\widehat{hh'})|\phi_{\mathbf{x},i,\mathbf{k}}^{r}\rangle \tag{28}$$

for $h, h' \in \mathcal{G}_{\mathbf{x}}$ and

$$\hat{t}_{\mathbf{R}}|\phi_{\mathbf{x},i,\mathbf{k}}^{r}\rangle = |\phi_{\mathbf{x},i,\mathbf{k}}^{r}\rangle e^{-i\mathbf{k}\cdot\mathbf{R}} \tag{29}$$

for $t_{\mathbf{R}} \in T$. The orbitals at other sites of $L_{\mathbf{x}}$ are then defined by $|\phi_{\mathbf{x}_\sigma,i,\mathbf{k}}^{r}\rangle \equiv \hat{g}_\sigma|\phi_{\mathbf{x},i,p_\sigma^{-1}\mathbf{k}}^{r}\rangle$. As before, $z_{g,g'} = \pm 1$ is a factor system of the projective representation originating from the spin degrees of freedom.

The choice of the position $\mathbf{x}$ to start with and the choice of an irrep $u_{\mathbf{x}}^{r}$ of the site symmetry group $\mathcal{G}_{\mathbf{x}}$ will specify an AI and its representation contents as we will see now.

Representation contents of an AI

To determine the transformation of $\{|\phi_{\mathbf{x}_\sigma,i,\mathbf{k}}^{r}\rangle\}_{\sigma,i,\mathbf{k}}$ under $\mathcal{G}$, note that any element $g \in \mathcal{G}$ can be uniquely decomposed as $g = t_{\mathbf{R}}g_\sigma h$ where $t_{\mathbf{R}} \in T$ and $h \in \mathcal{G}_{\mathbf{x}}$. In particular, we can decompose gg_σ as $t_{\mathbf{R}}g_{\sigma'}h$ with $\mathbf{R} = g(\mathbf{x}_\sigma) - \mathbf{x}_{\sigma'}$. Therefore,

$$\begin{aligned}
&\hat{g}|\phi_{\mathbf{x}_\sigma,i,\mathbf{k}}^{r}\rangle \\
&= z_{g,g_\sigma}(\widehat{gg_\sigma})|\phi_{\mathbf{x},i,p_\sigma^{-1}\mathbf{k}}^{r}\rangle \\
&= z_{g,g_\sigma}(\widehat{t_{\mathbf{R}}g_{\sigma'}h})|\phi_{\mathbf{x},i,p_\sigma^{-1}\mathbf{k}}^{r}\rangle \\
&= \frac{z_{g,g_\sigma}}{z_{g_{\sigma'},h}}\hat{t}_{\mathbf{R}}\hat{g}_{\sigma'}(\hat{h}|\phi_{\mathbf{x},i,p_\sigma^{-1}\mathbf{k}}^{r}\rangle) \\
&= \frac{z_{g,g_\sigma}}{z_{g_{\sigma'},h}}\sum_{i'}(\hat{t}_{\mathbf{R}}|\phi_{\mathbf{x}_{\sigma'},i',p_g\mathbf{k}}^{r}\rangle)[u_{\mathbf{x}}^{r}(h)]_{i'i} \\
&= \sum_{\sigma',i',\mathbf{k}'}|\phi_{\mathbf{x}_{\sigma'},i',\mathbf{k}'}^{r}\rangle[U_{\mathbf{x}}^{r}(g)]_{\sigma'i'\mathbf{k}',\sigma i\mathbf{k}},
\end{aligned} \tag{30}$$

where

$$\begin{aligned}
&[U_{\mathbf{x}}^{r}(g)]_{\sigma'i'\mathbf{k}',\sigma i\mathbf{k}} \\
&= \delta'_{\mathbf{x}_{\sigma'},g(\mathbf{x}_\sigma)}\delta''_{\mathbf{k}',p_g\mathbf{k}}e^{-i\mathbf{k}'\cdot(g(\mathbf{x}_\sigma)-\mathbf{x}_{\sigma'})}\frac{z_{g,g_\sigma}}{z_{g_{\sigma'},h_{\sigma',\sigma}^{g}}}[u_{\mathbf{x}}^{r}(h_{\sigma',\sigma}^{g})]_{i'i},
\end{aligned} \tag{31}$$

$\delta'_{\mathbf{x}_1,\mathbf{x}_2} = 1$ only when $\mathbf{x}_1 = \mathbf{x}_2$ modulo a lattice vector, and

$$h_{\sigma',\sigma}^{g} \equiv g_{\sigma'}^{-1}t_{\mathbf{x}_{\sigma'}-g(\mathbf{x}_\sigma)}gg_\sigma. \tag{32}$$

Note that $h_{\sigma',\sigma}^{g} \in \mathcal{G}_{\mathbf{x}}$ when $\delta'_{\mathbf{x}_{\sigma'},g(\mathbf{x}_\sigma)} = 1$. An AI constructed from an irrep $u_{\mathbf{x}}^{r}$ of $\mathcal{G}_{\mathbf{x}}$ has a representation $U_{\mathbf{x}}^{r}$ of $\mathcal{G}$. Although the discussion here is similar to the derivation of the induced representation U^α of $\mathcal{G}$ starting from an irrep $u_{\mathbf{k}}^{\alpha}$ of $\mathcal{G}_{\mathbf{k}}$ in Supplementary Note 1, there is an important difference. That is, the representation $U_{\mathbf{x}}^{r}$ is in general reducible, unlike U^α which is always irreducible whenever $u_{\mathbf{k}}^{\alpha}$ is irreducible.

Let us focus on a particular $\mathbf{k}$ in BZ. The AI's representation of $\mathcal{G}_{\mathbf{k}}$ is immediately given by $U_{\mathbf{x}}^{r}$ by restricting $\mathcal{G}$ to $\mathcal{G}_{\mathbf{k}}$. In particular, its character is

$$\begin{aligned}
&\chi_{\mathbf{x},\mathbf{k}}^{r}(g) \equiv \mathrm{tr}[U_{\mathbf{x}}^{r}(g)] \\
&= \sum_{\sigma=1}^{|\mathcal{W}_{\mathbf{x}}|}\delta'_{\mathbf{x}_\sigma,g(\mathbf{x}_\sigma)}\,e^{-i\mathbf{k}\cdot(g(\mathbf{x}_\sigma)-\mathbf{x}_\sigma)}\frac{z_{g,g_\sigma}}{z_{g_\sigma,h_{\sigma,\sigma}^{g}}}\chi_{\mathbf{x}}^{r}(h_{\sigma,\sigma}^{g}),
\end{aligned} \tag{33}$$

where $\chi_{\mathbf{x}}^{r}(h) \equiv \mathrm{tr}[u_{\mathbf{x}}^{r}(h)]$. Note that this result does not depend on the choice of $\mathbf{x}_\sigma$; even if one chose $\mathbf{x}'_\sigma = \mathbf{x}_\sigma + \mathbf{R}_\sigma$ instead, the character is unchanged.

Let $\chi_{\mathbf{k}}^{\alpha}(g) = \mathrm{tr}[u_{\mathbf{k}}^{\alpha}(g)]$ be the character of an irrep $u_{\mathbf{k}}^{\alpha}$ of $\mathcal{G}_{\mathbf{k}}$. Then, the irrep $u_{\mathbf{k}}^{\alpha}$ appears in $U_{\mathbf{x}}^{r}(g)$

$$n_{\mathbf{k}}^{\alpha} = \sum_{g\in\mathcal{G}_{\mathbf{k}}/T}\frac{1}{|\mathcal{G}_{\mathbf{k}}/T|}[\chi_{\mathbf{k}}^{\alpha}(g)]^*\chi_{\mathbf{x},\mathbf{k}}^{r}(g) \in \mathbb{Z}_{\geq 0} \tag{34}$$

times. This is the formula that gives $\mathbf{n}$ for a AI in general.

The general position

As a special case of the above general discussion, let us assume that the site symmetry group $\mathcal{G}_{\mathbf{x}}$ is trivial, i.e., $\mathcal{G}_{\mathbf{x}} = \{e\}$. In other words, let us assume that $\mathbf{x}$ belongs to the general Wyckoff position. There is only one trivial representation $u_{\mathbf{x}}^{r=1}(e) = 1$ for such a generic position. In this case, Supplementary Eq. (33) reduces to

$$\chi_{\mathbf{x},\mathbf{k}}^{r=1}(g) = |\mathcal{G}/T|\delta_{g,e}. \tag{35}$$

Therefore, using $\chi_{\mathbf{k}}^{\alpha}(e) = \mathrm{tr}[u_{\mathbf{k}}^{\alpha}(e)] = \dim[u_{\mathbf{k}}^{\alpha}]$, we get

$$n_{\mathbf{k}}^{\alpha}|_{\text{generic position}} = \frac{|\mathcal{G}/T|}{|\mathcal{G}_{\mathbf{k}}/T|}\dim[u_{\mathbf{k}}^{\alpha}] \geq 1. \tag{36}$$

Namely, the AI constructed from the trivial orbital on a generic position $\mathbf{x}$ contains every irrep $u_{\mathbf{k}}^{\alpha}$ at least once at each $\mathbf{k}$.

The special position

Next, consider a position $\mathbf{x}$ with a nontrivial $\mathcal{G}_{\mathbf{x}} > e$. In other words, $\mathbf{x}$ belongs to a special Wyckoff position. In this case, there are several irreps $u_{\mathbf{x}}^{r}$. We have a sum-rule separately for each $\mathbf{x}$:

$$n_{\mathbf{k}}^{\alpha}|_{\text{generic position}} = \sum_{r:\text{all irreps on }\mathbf{x}} \dim[u_{\mathbf{x}}^{r}]\; n_{\mathbf{k}}^{\alpha}|_{\text{irrep }u_{\mathbf{x}}^{r}\text{ on }\mathbf{x}}. \tag{37}$$

TR invariant AIs

To construct a TR invariant AI, we have to determine if the time-reversal (TR) symmetry $\mathcal{T}$ enhances the degeneracy of an irrep $u_{\mathbf{x}}^{r}$. This can be easily done by the method reviewed in Supplementary Note 1. Namely, one should compute the following sum, which can be either $+1$, 0, or -1.

$$\eta_{\mathcal{T}}\frac{1}{|\mathcal{G}_{\mathbf{x}}|}\sum_{h\in\mathcal{G}_{\mathbf{x}}} z_{h,h}\,\chi_{\mathbf{x}}^{r}(h^2) = \begin{cases} +1 & : u_{\mathbf{x}}^{r} \text{ by itself is TR invariant.} \\ -1 & : \text{Two } u_{\mathbf{x}}^{r}\text{'s are paired under TR.} \\ 0 & : u_{\mathbf{x}}^{r} \text{ and } (u_{\mathbf{x}}^{r})^{*} \text{ are different and are paired.} \end{cases} \tag{38}$$

Here, $\chi_{\mathbf{x}}^{r}(h) \equiv \mathrm{tr}[u_{\mathbf{x}}^{r}(h)]$ and $\eta_{\mathcal{T}} = -1$ ($\eta_{\mathcal{T}} = +1$) for the spinful (spinless) fermions. If the sum is either -1 or 0, the AI constructed from the irrep $u_{\mathbf{x}}^{r}$ on the site $\mathbf{x}$ alone is not TR symmetric, and one has to make a proper stacking with its TR pair.

Uniform basis

So far we have presented two constructions of a representation of $\mathcal{G}$: In Supplementary Note 1 we constructed one from an irrep $u_{\mathbf{k}}^{\alpha}$ of $\mathcal{G}_{\mathbf{k}}$, and the present note gives another one from an irrep $u_{\mathbf{x}}^{r}(h)$ of $\mathcal{G}_{\mathbf{x}}$. There is yet another construction of a representation of $\mathcal{G}$. Suppose that we know a representation $v(p_g)$ of the point group $\mathcal{G}/T$. Then a representation of $\mathcal{G}$ is given by

$$[U(g)]_{\sigma' i' \mathbf{k}', \sigma i \mathbf{k}} = \delta'_{\mathbf{x}_{\sigma'}, g(\mathbf{x}_{\sigma})}\, \delta''_{\mathbf{k}', p_g \mathbf{k}} e^{-i\mathbf{k}'\cdot(g(\mathbf{x}_{\sigma}) - \mathbf{x}_{\sigma'})} [v(p_g)]_{i' i}. \tag{39}$$

The advantage of this representation is that we have the same representation $v(p_g)$ on every site. As a result, the effect of spin-orbit coupling, for example, can be interpreted much easily than $U_{\mathbf{x}}^{r}$. Our leSM example discussed in the main text is formulated using this representation. The drawback is that this construction generally requires as input particular representations of $\mathcal{G}_{\mathbf{x}}$, in contrast to the earlier construction which applies to any representation, in particular to the irreducible ones.

Supplementary Note 3. Structure of {BS} and Computation of $X_{\rm BS}$

Mathematical details

Here, we discuss some relatively formal aspects of our mathematical framework. We will start with the claim

$$\{{\rm BS}\} \equiv \ker \mathcal{C} \cap \mathbb{Z}^D \;\Rightarrow\; \{{\rm BS}\} \simeq \mathbb{Z}^{d_{\rm BS}}, \qquad (40)$$

where $d_{\rm BS} \equiv \dim \ker \mathcal{C}$. This claim can be interpreted geometrically: Embedded in $\mathbb{R}^D$, $\mathbb{Z}^D$ is a hypercubic lattice and $\ker \mathcal{C}$ defines a $d_{\rm BS}$-dimensional hyperplane. {BS} is then the collection of lattice sites sliced by $\ker \mathcal{C}$, which defines a Bravais lattice in $d_{\rm BS}$ dimensions.

Alternatively, this can also be understood algebraically, as we now discuss in details. First observe $\{{\rm BS}\} \leq \mathbb{Z}^D$. Since any subgroup of a finitely generated abelian group is again finitely generated, and that no element in $\mathbb{Z}^D$ has finite order, we see that $\{{\rm BS}\} \simeq \mathbb{Z}^d$ for some $d \leq D$. Next, note that as $\mathcal{C}$ is a matrix of integer coefficients, its solution space can be identified as a vector subspace of $\mathbb{Q}^D$ (instead of $\mathbb{R}^D$). Let $\{\mathbf{q}_i \;:\; i = 1, \ldots, d_{\rm BS}\}$ be any complete basis for $\ker \mathcal{C} \simeq \mathbb{Q}^{d_{\rm BS}}$. Since only a finite number of rational numbers are involved, we can always multiply the basis by the least-common multiple of all the denominators to arrive at an integer-valued basis. This implies {BS} has at least $d_{\rm BS}$ linearly-independent elements, i.e. $d \geq d_{\rm BS}$. Now also observe that as $\{{\rm BS}\} \leq \ker \mathcal{C}$, $\ker \mathcal{C}$ has at least d linearly-independent vectors, which implies $d_{\rm BS} \leq d \leq d_{\rm BS}$.

Next we discuss the computation of $X_{\rm BS} \equiv \{{\rm BS}\}/\{{\rm AI}\}$, where $\{{\rm AI}\} \simeq \mathbb{Z}^{d_{\rm AI}} \leq \{{\rm BS}\}$ denotes the subgroup of BS arising from AIs. The first step in the analysis is to compare their ranks. $d_{\rm BS}$, a property of $\mathcal{C}$, is determined once all compatibility relations are found. $d_{\rm AI}$ can be computed as follows: Any AI can be understood as a stack of those arising from fully occupying an irrep of the site-symmetry group of a Wyckoff position. Hence, by focusing on the finite number of AIs arising from these irreps, we can find a (generally over-complete) basis for {AI} using the formalism developed in Supplementary Note 1 and Supplementary Note 2. Finally we simply extract the number of linearly independent combinations among them, which is by definition $d_{\rm AI}$.

Generally, we have $d_{\rm AI} \leq d_{\rm BS}$, and the general structure of the quotient group is given by

$$X_{\rm BS} = \mathbb{Z}^{d_{\rm BS} - d_{\rm AI}} \times \mathbb{Z}_{s_1} \times \mathbb{Z}_{s_2} \times \cdots \times \mathbb{Z}_{s_{d_{\rm AI}}}. \qquad (41)$$

It remains to compute the integers $s_i \geq 1$. In more physical terms, any element of $X_{\rm BS}$ corresponds to a class of BSs that cannot be obtained from integer combinations (i.e. stacking) of entries in {AI}. Now consider a $\mathbf{b} \in \{{\rm BS}\}$ with its equivalence class $[\mathbf{b}]$ being the generator of a factor Z_{s_i} in $X_{\rm BS}$. From definition, $s_i[\mathbf{b}] = [s_i \mathbf{b}]$ is the trivial element of $X_{\rm BS}$, i.e. $s_i \mathbf{b}$ is an AI. Running the argument in reverse, the torsion (i.e. finite-order) elements of $X_{\rm BS}$ correspond to (the classes of) fractions of AIs that are nonetheless in {BS}, and hence to compute the s_i's in Supplementary Eq. (41) one simply studies the possible set of coefficients for which $\sum_{i=1}^{d_{\rm AI}} q_i \mathbf{a}_i$, $q_i \in \mathbb{Q}$, is integer-valued. This can be readily computed using the Smith normal form, which is, loosely speaking, an integer-valued version of the singular-value decomposition (in our context).

An interesting observation is that, for all the 230×4 cases we studied, corresponding to all the SGs assuming spinless or spinful fermions with or without TR symmetry, we found $d_{\rm BS} = d_{\rm AI}$. This implies a basis for {BS} can be obtained by a suitable combination of the basis of {AI} using rational coefficients, which are found in the computation of s_i described above. Equivalently, we found that a BS can always be expanded as

$$\text{BS} = \sum_{\mathbf{x}} \sum_{r} q_{\mathbf{x},r}\, \mathbf{n}_{\mathbf{x},r}, \qquad (42)$$

where $\mathbf{n}_{\mathbf{x},r}$ denotes the $\mathbf{n}$ of the AI arising from fully occupying a site-symmetry group irrep $u_{\mathbf{x}}^r$ of the position $\mathbf{x}$ (it is sufficient to choose one representative of $\mathbf{x}$ for each Wyckoff position), and $q_{\mathbf{x},r} \in \mathbb{Q}$ are rational numbers. This is very similar to the decomposition of energy bands at high-symmetry momenta into irreps of the little group, except that this is now performed in a global manner over the BZ. An interesting open question is whether there is any symmetry setting, say when one studies the remaining magnetic SGs, for which $d_{\rm BS} > d_{\rm AI}$ and therefore leads to an infinite $X_{\rm BS}$ (i.e. some BSs remain non-atomic no matter how many copies we take). Alternatively, if such equality always holds for any symmetry settings, there should be a more elegant method to prove that $X_{\rm BS}$ is always finite.

In closing, we comment that the expansion in Supplementary Eq. (42) is similar in spirit to the notion of elementary energy bands developed in a series of work by Refs. [3–5]. In our language, these earlier results focus on such a decomposition between the AIs, and whether or not the building blocks of such decomposition, dubbed elementary energy bands, can be split into energy bands of lower fillings. These earlier works approached the problem from a real-space perspective, which in our language is about the structure of {AI}. In contrast, our formalism, centered on the structure of {BS}, automatically captures all the momentum-space constraints and is more suited for studying topological band structures: our notion of a global decomposition allows for quantum interference in momentum space, which is more general than that in Refs. [3–5]. Finally, we also remark that the results concerning energy band connectivity in Refs. [3–5]. has interesting implications on finding leSMs (specifically, for spinless fermions with TR symmetry).

(Note: From a more mathematical perspective, the earlier results in Refs. [3–5] are concerned with a decomposition in terms of the direct sum $\oplus$, whereas we have

first made a generalization $\oplus \to +$, akin to how representation rings are constructed, and then further assert that the decomposition is physically meaningful with rational coefficients, as long as the resulting (formal) sum lies in $\{\text{BS}\}$.)

Physical relevance of X_{BS}

Having discussed the mathematical aspect of the formalism, we now comment on why such notions are relevant to physical band structures. Recall we have motivated the definition of $\{\text{BS}\}$ by asserting that, as long as only symmetry properties are concerned, a set of energy bands can be labelled simply by a count of the multiplicities of each irrep at the high-symmetry momenta. A priori, it is insensible to say that an irrep appears a negative number of times. As mentioned in the main text, this leads to an additional condition in connecting the entries of $\{\text{BS}\}$ to physical band structures–for $\mathbf{n} \in \{\text{BS}\}$ to be physical, all components of $\mathbf{n}$ must be non-negative. Equivalently, one can define a physical subset

$$\{\text{BS}\}_{\text{P}} \equiv \{\text{BS}\} \cap \mathbb{Z}^D_{\geq 0} \subset \{\text{BS}\}. \quad (43)$$

As we have explained, all elements in $\{\text{BS}\}_{\text{P}}$ enjoy the properties of a general element in $\{\text{BS}\}$, say the decomposition in Supplementary Eq. (42). Therefore, the inclusion of unphysical entires, crucial for the group properties of $\{\text{BS}\}$, should be viewed merely as a mathematical way to simplify the analysis of the physical problem. (If one insists, one can identify $\{\text{BS}\}_{\text{P}}$ as a commutative monoid enjoying similar properties as the group $\{\text{BS}\}$. However, we do not find this perspective particularly useful in our discussion, and would rather stick with $\{\text{BS}\}$, a simpler mathematical gadget.)

However, there are still two points we have to establish in order in order to quantify the relevance of our mathematical framework to the study of real, physical band structures. First, we argue that all entries of $\{\text{BS}\}_{\text{P}}$ correspond to physically realizable band structures. To see this, consider an arbitrary element of $\{\text{BS}\}_{\text{P}}$, and let a set of physical bands possess the desired irrep content at all high-symmetry momentum points. (If only high-symmetry lines or planes are present, we choose arbitrary, isolated representatives.) This is always possible by a suitable arrangement of the energies of the irreps at these isolated momentum points. By symmetries and continuity, all compatibility relations are locally satisfied near these points. Suppose there are still certain band crossings obstructing us from identifying this set of bands as a BS. Since any pair of bands carrying the same symmetry representation will generically anti-cross, these band crossings must correspond to an exchange of irreps between our target set of bands and the others. As all compatibility relations are globally satisfied by assumption, such exchange must be accidental in nature, i.e. it is possible to perturb the Hamiltonian in a symmetric fashion to get rid of all the band crossings. This then leads to a BS corresponding to the specified element in $\{\text{BS}\}_{\text{P}}$.

Note that, however, there is a subtlety in the statement on generic anti-crossing: Topological band degeneracies, like Weyl points, can only be pushed away but not lifted, unless their topological charges are neutralized by their partners. This does not concern us, since by our definition of BS we will only be interested in band gaps at high-symmetry momenta, and therefore as long as these degeneracies can be moved away they do not affect our discussion. This also explains why the notion of reSM, which cannot be insulating due to the specified representation content, is still consistent with our notion of BS.

Second, we show that all nontrivial classes in X_{BS} have physical representatives. Let $\mathbf{b}$ be a representative of a nontrivial class in X_{BS} which is not physical, i.e. certain entries in $\mathbf{b}$ are negative. Now, we consider a small corollary from Supplementary Note 2: all irreps appear at least once in the AI corresponding to the generic Wyckoff position [see Supplementary Eq. (36)]. Therefore, we can always stack $\mathbf{b}$ with a sufficiently large number of copies of the generic AI and rectify the representation content. By definition, such stacking leads to a physical BS belonging to the same nontrivial class as $\mathbf{b}$, and hence all classes of X_{BS} have physical representatives.

Lower dimensional systems

Here we discuss X_{BS} for (quasi-)1D and 2D systems. As far as spinful electrons are concerned, the fact that lower dimensional systems in reality are embedded in the 3D space cannot be neglected, since the electronic spin degree of freedom is a projective representation of $O(3)$, the rotation of the 3D space. For brevity we will focus on 2D systems but the 1D case can be discussed in the same way.

The symmetry groups for 2D lattices lying in the 3D space are called layer groups. An element h of a layer group $\mathcal{L}$ maps (x, y, z) to (x', y', z'), where $(x', y') = q_h(x, y) + \mathbf{s}_h$ (q_h is a $O(2)$ matrix and $\mathbf{s}_h$ is a two component vector) and $z' = \xi_h z$ ($\xi_h = \pm 1$). The translation subgroup $T_{2\text{D}}$ of $\mathcal{L}$ is a group of lattice translations in the 2D plane, giving rise to the 2D crystal momentum (k_x, k_y). (At this moment k_z is not defined.) One can follow the same steps as in 3D to define $\{\text{BS}^{\mathcal{L}}\}$ and $\{\text{AI}^{\mathcal{L}}\}$, and $X^{\mathcal{L}}_{\text{BS}} = \{\text{BS}^{\mathcal{L}}\}/\{\text{AI}^{\mathcal{L}}\}$ for a layer group $\mathcal{L}$.

To make use of our results established for space groups, let us consider the space group $\mathcal{G}$ corresponding to a layer group $\mathcal{L}$, which is simply the layer group $\mathcal{L}$ endowed with a lattice translation T_z along z. More precisely, $\mathcal{G}$ is given by the semi-direct product of $\mathcal{L}$ and T_z, whose element $g = (h, t) \in \mathcal{G}$ ($h \in \mathcal{L}$ and $t \in T_z$) maps (x, y, z) to (x', y', z'), where $(x', y') = q_h(x, y) + \mathbf{s}_h$ and $z' = \xi_h z + t$. The product of $g = (h, t) \in \mathcal{G}$ and $g' = (h', t') \in \mathcal{G}$ is defined as $gg' = (hh', t + \xi_h t')$. We list the corresponding space group $\mathcal{G}$ for each layer group $\mathcal{L}$ in Supplementary

Table 1. Note that there is one different entry when our table is compared to the one provided in Supplementary Ref. [6]: we found that the correct correspondence for layer group 35 should be space group 38. To avoid confusion, we use the notations $\{\mathrm{BS}^{\mathcal{G}}\}$, $\{\mathrm{AI}^{\mathcal{G}}\}$, and $X_{\mathrm{BS}}^{\mathcal{G}} = \{\mathrm{BS}^{\mathcal{G}}\}/\{\mathrm{AI}^{\mathcal{G}}\}$ for a space group $\mathcal{G}$ in this section.

Given a layer group $\mathcal{L}$ and the corresponding space group $\mathcal{G}$, we expect $X_{\mathrm{BS}}^{\mathcal{G}}$ to encapsulate that of $X_{\mathrm{BS}}^{\mathcal{L}}$: stacking lower-dimensional nontrivial phases by translation symmetries will naturally give rise to weak topological phases, which are nontrivial as long as the translation symmetries remain intact, i.e., a nontrivial BS of $\mathcal{L}$ will never become trivial when lifted to $\mathcal{G}$ upon stacking. Generally, we also expect $X_{\mathrm{BS}}^{\mathcal{G}}$ to be richer than $X_{\mathrm{BS}}^{\mathcal{L}}$, since certain strong phases should become possible. These observations can be summarized by asserting the subgroup relation $X_{\mathrm{BS}}^{\mathcal{L}} \leq X_{\mathrm{BS}}^{\mathcal{G}}$(we will explain shortly the precise meaning of this symbolic relation). In the following, we formalize these observations and provide a (technical) proof for this relation. Before we dwell into the technical details, we remark that, given this natural subgroup relation, the finiteness of $X_{\mathrm{BS}}^{\mathcal{G}}$ implies that of $X_{\mathrm{BS}}^{\mathcal{L}}$, and therefore $X_{\mathrm{BS}}^{\mathcal{L}}$ can be readily computed using only the data on AI without finding and solving the compatibility relations.

Let us introduce a group homomorphism $f : \{\mathrm{BS}^{\mathcal{L}}\} \to \{\mathrm{BS}^{\mathcal{G}}\}$ through stacking of layers. Namely, starting from a given $\mathbf{b} \in \{\mathrm{BS}^{\mathcal{L}}\}$ of a layer group $\mathcal{L}$, we can get a band structure of $\mathcal{G}$ by stacking infinite copies of $\mathbf{b}$ in the z direction. To make this idea more concrete, let us take a tight-binding model $\hat{H}_{\mathcal{L}}$ symmetric under $\mathcal{L}$ such that the lowest ν bands of $\hat{H}_{\mathcal{L}}$ are isolated from other bands by a band gap at all high-symmetry points of the 2D BZ, and the combination of irreps of the lowest ν bands precisely agrees with $\mathbf{b}$. Given $\hat{H}_{\mathcal{L}}$, one can generate a $\mathcal{G}$ symmetric tight-binding model by

$$\hat{H}_{\mathcal{G}} = \sum_{t \in T_z} \hat{t} \hat{H}_{\mathcal{L}} \hat{t}^{-1}. \tag{44}$$

Since there is no inter-layer hopping, the band structure of $\hat{H}_{\mathcal{G}}$ is completely flat as a function of k_z, and as a result, the lowest ν bands remain isolated from other bands by a band gap at all high-symmetry points of the 3D BZ. This band structure of $\hat{H}_{\mathcal{G}}$ defines $f(\mathbf{b}) \in \{\mathrm{BS}^{\mathcal{G}}\}$. By construction, the homeomorphism $f(\mathbf{b}_1 + \mathbf{b}_2) = f(\mathbf{b}_1) + f(\mathbf{b}_2)$ is obvious. Furthermore, f maps $\mathbf{a} \in \{\mathrm{AI}^{\mathcal{L}}\}$ to $f(\mathbf{a}) \in \{\mathrm{AI}^{\mathcal{G}}\}$. Therefore, f defines a group homomorphism

$$f : X_{\mathrm{BS}}^{\mathcal{L}} \to X_{\mathrm{BS}}^{\mathcal{G}} \tag{45}$$

Below we show that this f is injective. If this is the case, $\tilde{X}_{\mathrm{BS}}^{\mathcal{L}} \equiv \mathrm{Im} f$ is isomorphic to $X_{\mathrm{BS}}^{\mathcal{L}}$ and is a subgroup of $X_{\mathrm{BS}}^{\mathcal{G}}$.

To this end, let us introduce a projection p that defines a homomorphism from $\{\mathrm{BS}^{\mathcal{G}}\}$ to $\{\mathrm{BS}^{\mathcal{L}}\}$. Given $\mathbf{B} \in \{\mathrm{BS}^{\mathcal{G}}\}$, one can project out all entries of $\mathbf{B}$ associated with high-symmetry points with $k_z \neq 0$, keeping only entries associated with high-symmetry points with $k_z = 0$. By definition $p(\mathbf{B})$ satisfies all compatibility conditions imposed on the irreps of $\mathcal{L}$ and hence is an element of $\{\mathrm{BS}^{\mathcal{L}}\}$. The projection p in fact acts as an inverse of f: $p(f(\mathbf{b})) = \mathbf{b}$. Furthermore, p maps $\mathbf{A} \in \{\mathrm{AI}^{\mathcal{G}}\}$ to $p(\mathbf{A}) \in \{\mathrm{AI}^{\mathcal{L}}\}$. This can be seen by the fact that, for every Wyckoff position of $\mathcal{G}$, there exists a Wyckoff position of $\mathcal{L}$ that is either identical, or differs only by the value of z. The difference of z does not affect $p(\mathbf{A})$ since the projection p sets $k_z = 0$. Given these properties of p, it is easy to prove that $\ker f = \{e\}$. If not, there must be a $\mathbf{b} \in \{\mathrm{BS}^{\mathcal{L}}\}$ belonging to a nontrivial class of $X_{\mathrm{BS}}^{\mathcal{L}}$ which is mapped to $\mathbf{A} \in \{\mathrm{AI}^{\mathcal{G}}\}$ by f. Then $\mathbf{b} = p(\mathbf{A}) \in \{\mathrm{AI}^{\mathcal{L}}\}$ is an AI, contradicting with the assumption that $\mathbf{b}$ is nontrivial. Hence the proof.

Supplementary Table 1. Correspondence between space groups and layer groups.

LG	SG	LG	SG	LG	SG	LG	SG
1	1	21	18	41	51	61	123
2	2	22	21	42	53	62	125
3	3	23	25	43	54	63	127
4	6	24	28	44	55	64	129
5	7	25	32	45	57	65	143
6	10	26	35	46	59	66	147
7	13	27	25	47	65	67	149
8	3	28	26	48	67	68	150
9	4	29	26	49	75	69	156
10	5	30	27	50	81	70	157
11	6	31	28	51	83	71	162
12	7	32	31	52	85	72	164
13	8	33	29	53	89	73	168
14	10	34	30	54	90	74	174
15	11	35	38	55	99	75	175
16	13	36	39	56	100	76	177
17	14	37	47	57	111	77	183
18	12	38	49	58	113	78	187
19	16	39	50	59	115	79	189
20	17	40	51	60	117	80	191

LG: Layer Group; SG: Space Group.

Supplementary Note 4. Filling-enforced Quantum Band Insulators

In this note we provides details on the feQBIs found in this work, which we briefly mentioned in the main text. A band insulator, invariant under a set of symmetries (including an SG $\mathcal{G}$), is called a feQBI if the number of the occupied bands (i.e., the filling) is different from that of any AIs with the same symmetry. Thus feQBIs are a special case of reQBIs. As all possible TR-symmetric feQBIs have been discussed in Supplementary Ref. [14], we will mainly consider systems without TR invariance.

TR-breaking spinless filling-enforced quantum band insulators

Let us start with feQBIs in the system of spinless fermions, which necessarily break the TR symmetry. This is because of the following reason: If there existed a TR-symmetric feQBI for spinless fermions, we could immediately construct a TR-symmetric feQBI for spinful fermions with the spin SU(2) symmetry. However, we know that the latter does not exist according to Supplementary Ref. [14].

As a strategy to find feQBIs, one can focus on the electron fillings among elements of $\{\text{BS}\}$ and $\{\text{AI}\}$. Whenever there is a mismatch between them, it indicates that certain nontrivial element of X_{BS} can be diagnosed simply using the electron filling. This question can again be tackled efficiently using the vector-space like structure of $\{\text{BS}\}$ and $\{\text{AI}\}$. We indeed found a mismatch for several SGs for spinless fermions without TR symmetry.

The mismatch can possibly occur only in those 12 SGs listed in Supplementary Table **2**. To see this, we should focus on (the maximal) fixed-point-free subgroups Γ of $\mathcal{G}$ (not to be confused with the Γ point in BZ). For a fixed-point-free SG Γ, it is easy to show that a certain number of bands must cross with each other somewhere at high-symmetry points or on high-symmetry lines, and that the BI fillings are integer multiples of $\nu_\Gamma > 1$. Hence, if $\mathcal{G}$ contains Γ as a subgroup, we know that any $\mathcal{G}$-symmetric BI should have a filling $\nu_\Gamma n$. For all 218 SGs not listed in Supplementary Table **2**, there exists a $\mathcal{G}$-symmetric AI with the filling ν_Γ. Hence, there cannot be any mismatch of the filling between $\{\text{BS}\}$ and $\{\text{AI}\}$ for them. On the other hand, for 8 SGs out of the 12 SGs in Supplementary Table **2**, we found that the filling of the elements of $\{\text{BS}\}$ is $2\mathbb{Z}$, while that of $\{\text{AI}\}$ is $4\mathbb{Z}$. Hence we can expect a feQBI at filling $\nu = 4n + 2$.

The case of SG 220 is more nontrivial. In Supplementary Table **2**, we show the set of fillings $\mathcal{S}_{\mathcal{G}}^{\text{AI}}$ ($\mathcal{S}_{\mathcal{G}}^{\text{BI}}$) that correspond to at least one AI (BI). This set was computed by the following way. Let $\nu_{\mathbf{x},r}$ be the filling of the AI arising from fully occupying a site-symmetry group irrep $u_{\mathbf{x}}^r$ of the position $\mathbf{x}$. Then we take superpositions with non-negative integer coefficients: $\mathcal{S}_{\mathcal{G}}^{\text{AI}} = \{\sum_{\mathbf{x},r} m_{\mathbf{x},r}\nu_{\mathbf{x},r} \ : \ m_{\mathbf{x},r} \in \mathbb{Z}_{\geq 0}\}$, which is, in principle, different from the fillings for $\{\text{AI}\} \cap \mathbb{Z}_{\geq 0}^D$. On the other hand, $\mathcal{S}_{\mathcal{G}}^{\text{BI}}$ is simply the fillings for $\{\text{BI}\} \cap \mathbb{Z}_{\geq 0}^D$. In the case of $\mathcal{G} = 220$, although the filling for $\{\text{AI}\}$ and $\{\text{BS}\}$ are both $2\mathbb{Z}$, $\mathcal{S}_{\mathcal{G}}^{\text{AI}}$ and $\mathcal{S}_{\mathcal{G}}^{\text{BI}}$ do not agree with each other and there is a feQBI at filling $\nu = 4$:

$$\mathcal{S}_{\mathcal{G}}^{\text{AI}} = 2\mathbb{Z}_{\geq 0} \setminus \{2, 4, 10\} = \{0, 6, 8, 12, 14, 16, \ldots\}, \quad (46)$$

$$\mathcal{S}_{\mathcal{G}}^{\text{BI}} = 2\mathbb{Z}_{\geq 0} \setminus \{2\} = \{0, 4, 6, 8, 10, 12, 14, 16, \ldots\}. \quad (47)$$

Supplementary Table **3** summarizes TR breaking spinless feQBIs in $8 + 1 = 9$ SGs identified above. Note that these new feQBI examples are, in a sense, more intriguing than the ones we discussed in Supplementary Ref. [14]. In our previous examples assuming TR-symmetric spinful electrons, the filling condition was enriched by Kramers degeneracy, and feQBIs were discovered at odd-integer site fillings. In these new examples we discovered, however, the site filling is fractional for any choice of SG-symmetric lattices. Superficially, this might appear to be at odds with the conventional wisdom that gapped phases at fractional fillings are associated with either discrete symmetry breaking or intrinsic topological order–both only possible in the presence of interactions. Rather, these examples highlight the fact that spatial symmetries can lead to intrigue constraints between filling and phases, as was discussed in Supplementary Ref. [11], which, in fact, correctly predicted that symmetry-protected topological phases might be possible for these systems at such fractional fillings.

A tight-binding model

As we have cautioned before, a nontrivial BS can generally be either a reSM or a reQBI. To establish that the filling mismatch indeed leads to feQBIs, one has to further assert that a band gap is possible at all generic momenta in the BZ. To achieve this goal we explicitly constructed a tight-binding model for each of feQBIs in Supplementary Table **3**. As a concrete example, let us discuss the case of SG 106, which hosts feQBI at filling $\nu = 2$.

Consider a tight-binding model (4 band model) defined on $\mathcal{W}_b^{106}$ with one orbital per site. Here, $\mathcal{W}_b^{106}$ means the Wyckoff position with the Wyckoff letter b in Supplementary Ref. [2]:

$$(0, \tfrac{1}{2}, z), (\tfrac{1}{2}, 0, z + \tfrac{1}{2}), (\tfrac{1}{2}, 0, z), (0, \tfrac{1}{2}, z + \tfrac{1}{2}) \quad (48)$$

The site symmetry of this Wyckoff position is the π-rotation about the z-axis. We use a p orbital that flips sign under the rotation. These informations specify the representation $[U_{\mathbf{k}}(g)]_{\sigma' j, \sigma i} \equiv [U_{\mathbf{x}}^r(g)]_{\sigma' j \mathbf{k}', \sigma i \mathbf{k}}$ of $\mathcal{G} = 106$ in the tight-binding model as discussed in Supplementary Note 2. One can construct $U_{\mathbf{k}}(g)$ using Supplementary Eq. (31). (In this particular example, i, j la-

Supplementary Table 2. Band insulator fillings for some special space groups

Space group $\mathcal{G}$	$\mathcal{S}_{\mathcal{G}}^{\mathrm{AI}}$	$\mathcal{S}_{\mathcal{G}}^{\mathrm{BI}}$	$\mathcal{S}_{\mathcal{G}}^{\Gamma}$
106,110	$4\mathbb{Z}_{\geq 0}$	$2\mathbb{Z}_{\geq 0}$	$2\mathbb{Z}_{\geq 0}$
73,133,142, 206, 228	$4\mathbb{Z}_{\geq 0}$	$2\mathbb{Z}_{\geq 0} \setminus \{2\}$	$2\mathbb{Z}_{\geq 0}$
135	$4\mathbb{Z}_{\geq 0}$	$4\mathbb{Z}_{\geq 0}$	$2\mathbb{Z}_{\geq 0}$
199, 214	$2\mathbb{Z}_{\geq 0} \setminus \{2\}$	$2\mathbb{Z}_{\geq 0} \setminus \{2\}$	$2\mathbb{Z}_{\geq 0}$
220	$2\mathbb{Z}_{\geq 0} \setminus \{2, 4, 10\}$	$2\mathbb{Z}_{\geq 0} \setminus \{2\}$	$2\mathbb{Z}_{\geq 0}$
230	$4\mathbb{Z}_{\geq 0} \setminus \{4\}$	$2\mathbb{Z}_{\geq 0} \setminus \{2, 4\}$	$2\mathbb{Z}_{\geq 0}$

AI: Atomic Insulator; BI: Band Insulator; $\mathcal{S}_{\mathcal{G}}^{\mathrm{AI}}$: the set of fillings for AI; $\mathcal{S}_{\mathcal{G}}^{\mathrm{BI}}$: the set of fillings for BI; $\mathcal{S}_{\mathcal{G}}^{\Gamma}$: is the set of BI fillings for fixed-point free subgroup Γ of $\mathcal{G}$.
Note–By definition, we have $\mathcal{S}_{\mathcal{G}}^{\mathrm{AI}} \leq \mathcal{S}_{\mathcal{G}}^{\mathrm{BI}} \leq \mathcal{S}_{\mathcal{G}}^{\Gamma}$, and in fact $\mathcal{S}_{\mathcal{G}}^{\mathrm{AI}} = \mathcal{S}_{\mathcal{G}}^{\mathrm{BI}} = \mathcal{S}_{\mathcal{G}}^{\Gamma}$ for all 218 SGs not listed here.

Supplementary Table 3. Summary of results for the 12 space groups listed in Supplementary Table 2.

Space group $\mathcal{G}$	X_{BS}	Generator of X_{BS}
106,110	$\mathbb{Z}_2$	feQBI at filling 2
73,133,142, 206, 228	$\mathbb{Z}_2$	feQBI at filling 6
135	1	—
199, 214	1	—
220	$\mathbb{Z}_2$	feQBI at filling 4
230	$\mathbb{Z}_2$	feQBI at filling 6, 10

X_{BS}: the quotient group between the group of band structures and atomic insulators.

bel can be dropped since the orbital is a 1D representation). In this notation, $U_{\mathbf{k}}(g)$ satisfies multiplication rule $U_{p_{g_2}\mathbf{k}}(g_1)U_{\mathbf{k}}(g_2) = U_{\mathbf{k}}(g_1 g_2)$.

We construct a $\mathcal{G}$-symmetric Hamiltonian $H_{\mathbf{k}}$ in two steps: First, we choose an arbitrary 4 by 4 Hermitian matrix $h_{\mathbf{k}}$; next, we symmetrize $h_{\mathbf{k}}$ by performing the summation:

$$H_{\mathbf{k}} = \sum_{g \in \mathcal{G}/T} U_{\mathbf{k}}(g)^{\dagger} h_{p_g \mathbf{k}} U_{\mathbf{k}}(g). \tag{49}$$

After the summation, $H_{\mathbf{k}}$ automatically fulfills the symmetry requirement, i.e., $H_{p_g\mathbf{k}} U_{\mathbf{k}}(g) = U_{\mathbf{k}}(g) H_{\mathbf{k}}$.

To realize an example of feQBI for 106, the following choice of $h_{\mathbf{k}}$ works

$$h_{\mathbf{k}} = \Delta \begin{pmatrix} (\cos k_x - \cos k_y) & 1 & 0 & -i \\ 1 & 0 & 0 & 0 \\ 0 & 0 & 0 & -i \\ i & 0 & i & 0 \end{pmatrix}, \tag{50}$$

where Δ sets the energy scale of the problem. The symmetrized Hamiltonian $H_{\mathbf{k}}$ has a very large gap. At each $\mathbf{k}$, let $\delta E_{\mathbf{k}}$ be the band gap between the second and the third band and $W_{\mathbf{k}}$ be the band width (the energy difference between the lowest and the highest band). Then we found $\delta E = 0.33W$ where $\delta E = \min_{\mathbf{k}} \delta E_{\mathbf{k}}$ and $W = \max_{\mathbf{k}} W_{\mathbf{k}}$. An example band structure is plotted in Supplementary Fig. 1, which demonstrates that even at a filling of half an electron per site, a band gap is nonetheless possible. We also found that the Chern numbers of the tight-binding model is zero.

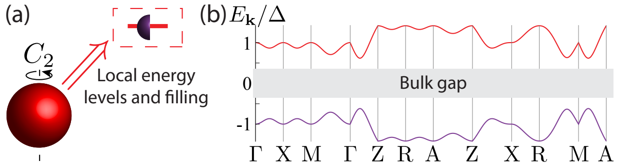

Supplementary Figure 1. Filling-enforced quantum band insulators in unconventional symmetry settings. Here, we focus on one such example found among spinless systems in the tetragonal space group 106 without time-reversal symmetry. (a) For this space group, a maximal-symmetry site (red sphere) is invariant under a C_2 rotation, and symmetries require that there are at least four such sites in the unit cell. We consider a filling of $\nu = 2$, corresponding to a site filling of 1/2. Note that, given the space group symmetries, any other choice of lattices correspond to site fillings $\leq 1/2$. (b) Nonetheless, a band insulator is possible at such filling, as shown in the plotted band structure. Each band shown is doubly-degenerate, but such degeneracy originates from nonessential additional symmetries in the simple tight-binding model we constructed, and therefore can be lifted.

In Supplementary Table 4, we list some possible symmetry settings for feQBIs in other SGs.

TR-symmetric filling-enforced quantum band insulators

Finally, we briefly comment on the implications of the present work on the feQBIs we introduced in Supplementary Ref. [14], which arise in systems with TR symmetry and significant spin-orbit coupling. There, the focus of study are SGs 199, 214, 220 and 230, which have

Supplementary Table 4. Possible symmetry setting for the filling-enforced quantum band insulators in Supplementary Table 3.

Space group	Wyckoff position	orbital	$m_{\rm tot}$	ν
106	b	p^*	4	2
110	a	p^*	4	2
133	$a+e$	s	12	6
220	c	s	8	4
228(>142, 73)	d	s	12	6
230(> 206)	a	$s+p^{**}$	16	6,10

feQBI: filling-enforced quantum band insulators; $m_{\rm tot}$: the total number of bands in this setting; ν: electron fillings per primitive unit cell for which feQBIs are possible;
* The site symmetry group for the Wyckoff position b of 106 and the Wyckoff position a of 110 are an order 2 group, and the p orbital refers to the representation with -1 for the non-identity element.
** The site symmetry group for the Wyckoff position a of 230 is $\bar{3}$ group, generated by the improper three-fold rotation IC_3. The p orbital refers to the one that has $e^{i\frac{2\pi}{6}}$ eigenvalue of IC_3.

an intriguing property in the ratios between the Wyckoff position multiplicities: They were dubbed Wyckoff-mismatched as some Wyckoff multiplicities are not integer multiples of the smallest one. In these systems, it was realized that QBIs are possible at non-atomic fillings, and hence the name feQBIs.

However, in the analysis of Supplementary Ref. [14] the mismatch between BSs and AIs is discussed in terms of their corresponding physical fillings, which as we have explained is a stronger condition than that exposed using only the abelian group structures of $\{\text{BS}\}$ and $\{\text{AI}\}$. As a result, these feQBIs could be trivial in $X_{\rm BS}$. This is indeed the case for some of them: both 199 and 214 have no nontrivial BSs in $X_{\rm BS} = \mathbb{Z}_1$. (By our convention, they are omitted from Supplementary Table III of the main text.)

It remains to study 220 and 230, which have $X_{\rm BS} = \mathbb{Z}_2$ and $\mathbb{Z}_4$ respectively. We found that for both cases, some feQBIs are again in the trivial class, i.e. they can be understood as integer combinations of AIs (but with negative coefficients, resulting in unrealizable AI upon enforcing the physical conditions). However, the nontrivial class for 220 can be represented by a feQBI filling $\nu = 20$. More interestingly, the entry $2 \in \mathbb{Z}_4$ for 230 can be represented by a feQBI at filling $\nu = 8$. Since 230 is centrosymmetric, as we have argued in the main text the generator $1 \in \mathbb{Z}_4$ has to be identified with the strong TI. This implies some $\nu = 8$ feQBIs in 230 realize the doubled strong TI phase we discussed in the main text, which has inversion-protected nontrivial entanglement signature, albeit no physical surface state is expected.

All in all, we found that generally the TR-symmetric feQBIs do not have any simple relationship with $X_{\rm BS}$, although there are indeed examples which are nontrivial from both perspectives.

Supplementary Note 5. Representation-enforced Quantum Band Insulators and Semimetals

General relation

As we have discussed in the main text, our notion of a BS is compatible with both band insulators and semimetals, as long as a continuous band gap is sustained at all high-symmetry momenta. In particular, the nontrivial entries in $X_{\rm BS}$ can sometimes correspond to reSM, which are guaranteed to be semimetallic due to the specification of the symmetry content. These systems are exemplified by the 3D systems with inversion but not TR symmetries [7, 8].

Since reSMs are, by definition, also diagnosable using the representation content, one can systematically isolate them from {BS}. However, it is important to realize that reSMs do not form a subgroup of {BS}, since stacking two of them (say) may lead to a band insulator [7, 8]. In contrast, it is guaranteed that stacking two band insulators will lead to yet another band insulator. This suggests that we should identify the subgroup $\{\rm BI\} \leq \{\rm BS\}$. Further observe $\{\rm AI\} \leq \{\rm BI\}$, it is natural to define the following further diagnosis of the elements in {BS}:

$$X_{\rm SM} \equiv \frac{\{\rm BS\}}{\{\rm BI\}}; \qquad X_{\rm BI} \equiv \frac{\{\rm BI\}}{\{\rm AI\}}. \tag{51}$$

The nontrivial entries in $X_{\rm SM}$ and $X_{\rm BI}$ respectively correspond to reSMs and reQBIs. As we have alluded to, the nontrivial elements in $X_{\rm BS}$ are either reSMs or reQBIs, and hence, unsurprisingly, the three objects $X_{\rm BS}$, $X_{\rm SM}$ and $X_{\rm BI}$ are not independent. From definitions, one can check that

$$X_{\rm SM} = \frac{X_{\rm BS}}{X_{\rm BI}}. \tag{52}$$

Or in a more formal language, $X_{\rm BS}$ can be viewed as a central extension of $X_{\rm SM}$ by $X_{\rm BI}$. Curiously, this extension is generally nontrivial. As a concrete example, consider the $\mathbb{Z}_4$ factor in $X_{\rm BS} = (\mathbb{Z}_2)^3 \times \mathbb{Z}_4$ for SG 2 (inversion only) assuming no TR symmetry. From Refs. [7, 8], we see that the generator of this factor is a reSM (with inversion related Weyl points), and the twice of that, corresponding to $2 \in \mathbb{Z}_4$, is a reQBI with a quantized magnetoelectric response of $\theta = \pi$. This shows that for this particular example,

$$X_{\rm BI} = (\mathbb{Z}_2)^4; \quad X_{\rm SM} = \mathbb{Z}_2, \tag{53}$$

and the $\mathbb{Z}_4$ factor in $X_{\rm BS}$ originates from the nontrivial extension of $\mathbb{Z}_2$ by $\mathbb{Z}_2$.

Representation-enforced quantum band insulators

As we will discuss in the following, given any symmetry setting one can systematically study all reSMs using symmetry arguments together with knowledge on the generic stability of Femri surfaces. For instance, for centrosymmetric systems with TR symmetry and significant spin-orbit coupling, stable band degeneracy must happen at a high-symmetry momentum, and therefore we can rule out the possibility of reSMs in these problems, i.e. for such settings we have $X_{\rm SM} = \mathbb{Z}_1$ and hence $X_{\rm BI} = X_{\rm BS}$. However, even after $X_{\rm BI}$ is obtained our approach is still based on symmetry-labels in nature, and therefore does not necessarily detect all nontrivial phases. An important future direction is to incorporate the tenfold way classification into our current framework [9], akin to the arguments given in Supplementary Ref. [10].

Representation-enforced semimetals

For a tight-binding model with inversion but not TR symmetry, some combinations of the parity eigenvalues at TRIMs predict the existence of Weyl points somewhere in the interior of the BZ [7, 8]. The semimetallic behavior of such systems is enforced by the specification of the representations, and we refer to such systems as reSMs. In this section, we ask whether similar phenomena occur for other SG, i.e., given an SG and a set of integers $\mathbf{n}$ for irreps at high-symmetry momenta, we ask if there are enforced gap closing somewhere at non-high-symmetry points in the BZ. For simplicity, in the following we answer this question for three-dimensional system without TR symmetry, such that the topologically-protected gap closing at non-high-symmetry momenta corresponds to Weyl points.

Let us consider a unit sphere S^2 around the Γ point of the BZ. The radius of the sphere is set to be much smaller than any reciprocal lattice vectors. If there are Weyl points at generic momenta in the BZ (i.e. not high-symmetry), we should be able to move them to the surface of this sphere without breaking any symmetry or changing the value of $\mathbf{n}$. Note that, in the following 'sphere' always refer to S^2, which does not include the interior of the sphere.

An SG element $g \in \mathcal{G}$ moves a point $\mathbf{k}$ on S^2 to $p_g\mathbf{k}$ on S^2. For this transformation, the translation part $\mathbf{t}_g$ of g does not show up at all and hence what is really important is the point group $\mathcal{P} \simeq \mathcal{G}/T$ of the SG, whose elements are the orthogonal matrices p_g ($g \in \mathcal{G}$). There are only 32 crystallographic point groups in 3D, and we will systematically discuss them in the following. One can introduce the notion of the little group, the symmetry orbit, and Wyckoff positions to this S^2 in the same way as before. Given $\mathbf{k}$ on S^2, the little group $\mathcal{G}_{\mathbf{k}}$ is defined as

$$\mathcal{G}_{\mathbf{k}} = \{p_g \in \mathcal{P} \ : \ p_g\mathbf{k} = \mathbf{k}\}. \tag{54}$$

The symmetry orbit of $\mathbf{k}$ (aka the star of $\mathbf{k}$) is defined as $\{p_g\mathbf{k} \ : \ p_g \in \mathcal{P}\}$. Also, two points $\mathbf{k}_1$ and $\mathbf{k}_2$ belong to

the same (momentum-space) Wyckoff position iff there exists $g \in \mathcal{G}$ such that $\mathcal{G}_{\mathbf{k}_2} = p_g \mathcal{G}_{\mathbf{k}_1} p_g^{-1}$. Finally, let us define the irreducible part (or the fundamental domain) F of the sphere. The fundamental domain F tessellates the S^2 under the action of $\mathcal{P}$. Namely, any point on the sphere can be uniquely represented as $p_g\mathbf{k}$, where $\mathbf{k} \in F$ and $p_g \in \mathcal{P}$.

With this preparation, let us discuss the stability of the Weyl points against symmetry preserving deformations. Suppose that the fundamental domain F contains a Weyl point with the chirality $+1$. (By assumption, generically this Weyl point does not sit at the boundary of F.) Then there must be at least $|\mathcal{P}|$ Weyl points on the sphere in total. The domain p_gF will contain a Weyl point with the chirality $\det[p_g]$. Suppose first that $\det[p_g] = +1$ for all $p_g \in \mathcal{P}$. This is the case for 11 out of 32 point groups in 3D. In this case, since the net chirality in the entire BZ must vanish, there must be another Weyl point in F with the chirality -1 (that belongs to a separate symmetry orbit), and the two Weyl points with the opposite chirality will freely annihilate with each other. This implies reSMs cannot exist for SGs with these point groups.

Next we discuss the remaining $32 - 11 = 21$ point groups, which always have half of the elements having $\det[p_g] = -1$. Again the number of symmetry-related Weyl points is $|\mathcal{P}|$, but now $|\mathcal{P}|/2$ of them has $+1$ chirality and the other half has the -1 chirality. A prioi, this could be compatible with a reSM, since the net Weyl charge in the entire BZ vanishes. However, this still requires further symmetry analysis. We ask if one can move Weyl points on S^2 and merge pairs of them with opposite chirality without breaking the SG symmetry or changing the number of irreps $\mathbf{n}$. We found that 18 of the 21 remaining point groups contain at least one mirror reflection symmetry and there exists a Wyckoff position on the sphere which is symmetric only under the mirror. For these point groups, two Weyl points with the opposite chirality should be able to annihilate at this Wyckoff position. Provided this is true, this again rules out reSMs in these settings. In the following, we will first assume the validity of this claim and study the remaining point groups; a more thorough analysis is left for future works.

The remaining three point groups are $\bar{1}$, $\bar{3}$, and $\bar{4}$ in the Hermann-Mauguin notation. Among them, $\bar{1}$ is the point group generated by the inversion symmetry, and we know that Weyl points can be protected by $\mathbf{n}$ in this case [7, 8]. $\bar{3}$ contains $\bar{1}$ as a subgroup, and therefore also contains the rotation C_3. Due to the relation between the C_3 eigenvalues and the Chern number (for any momentum plane perpendicular to the rotation axis) [13], one can show that the only reSMs for $\bar{3}$ are those that are diagnosed by the $\bar{1}$ subgroup. As such, the only possibly nontrivial candidate is thus the point group $\bar{4}$, which is the point group of the SG symmetry 81 and 82. We looked at 81 for the spinful but TR breaking setting, and indeed found an reSM. X_{BS} for 81 is $(\mathbb{Z}_2)^2 \times \mathbb{Z}_4$. The reSM is the generator of one of the two $\mathbb{Z}_2$ factor. In fact, this reSM in SG 81 can also be understood from the Chern number arguments in Supplementary Ref. [13].

In closing, we remark that the analysis of reSM has to be modified in other symmetry setting, since depending on symmetries topologically stable gaplessness may not be of the Weyl type. For instance, nodal lines are stable in time-reversal and inversion symmetric systems with negligible spin-orbit coupling, and therefore the argument above has to be modified from the motion of points to nodal rings. In addition, similar to the point-group symmetries we discussed, TR will also constrain the possible distribution of the gap closing, and therefore have to be taken into account in the analysis of reSMs in TR-symmetric system.

Supplementary Table 5. **Characterization of band structures for systems with significant spin-orbit coupling and no time-reversal symmetry.**

d	Space groups
1	1, 4, 7, 9, 16, 19, 22, 23, 25, 26, 27, 29, 33, 36, 38, 39, 42, 44, 45, 76, 78, 93, 101, 105, 109, 110<br>144, 145, 169, 170, 180, 181
2	8, 21, 31, 35, 37, 41, 43, 46, 80, 92, 94, 96, 97, 98, 102, 106, 107, 108, 161, 208, 214
3	5, 6, 18, 20, 30, 32, 34, 40, 48, 50, 56, 59, 61, 62, 68, 70, 73, 89, 99, 103, 146, 151, 152, 153, 154, 160<br>171, 172, 178, 179, 185, 186, 195, 196, 197, 198, 209, 210, 211, 212, 213
4	24, 28, 54, 57, 60, 72, 77, 90, 91, 95, 100, 104, 133, 137, 142, 155, 158, 159, 177, 183, 184, 199, 207
5	3, 14, 17, 52, 63, 64, 67, 79, 111, 112, 115, 116, 119, 120, 121, 126, 130, 134, 138, 156, 157, 173, 182
6	11, 15, 49, 69, 71, 113, 114, 117, 118, 125, 129, 132, 135, 141, 149, 150, 215, 216, 217, 218, 219
7	13, 51, 55, 66, 74, 122, 131, 136, 143, 167, 220, 228, 230
8	58, 65, 75, 88, 140, 163, 165, 222, 223, 224
9	2, 47, 53, 86, 139, 168, 201, 203, 205, 206, 227
10	12, 187, 189, 193, 194, 202, 204, 226
11	82, 85, 124, 148, 166, 200, 225, 229
12	81, 127, 128, 162, 164, 188, 190
13	84, 123, 147, 192
14	191, 221
15	10
16	87, 176
21	174
24	83
27	175

d: the rank of the abelian group formed by the set of band structures.

Supplementary Table 6. **Characterization of band structures for systems of spinless fermions without time-reversal symmetry.**

d	Space groups
1	1, 4, 7, 9, 19, 29, 33, 76, 78, 144, 145, 169, 170
2	8, 31, 36, 41, 43, 80, 92, 96, 110, 161
3	5, 6, 18, 20, 26, 30, 32, 34, 40, 45, 46, 61, 106, 109, 146, 151, 152, 153, 154, 160, 171, 172, 178, 179, 198, 212 213
4	24, 28, 37, 39, 60, 62, 77, 91, 95, 102, 155, 158, 159, 185, 186, 199, 210
5	3, 14, 17, 27, 42, 44, 52, 56, 57, 79, 94, 98, 101, 104, 108, 156, 157, 173, 182, 196, 197, 214
6	11, 15, 35, 38, 54, 70, 73, 100, 103, 105, 107, 149, 150, 184
7	13, 22, 23, 59, 64, 68, 90, 114, 122, 142, 143, 167, 180, 181, 195, 208, 209, 211, 220
8	21, 58, 63, 75, 88, 97, 113, 130, 137, 163, 165, 183, 219
9	2, 25, 48, 50, 53, 55, 72, 86, 99, 117, 118, 120, 133, 135, 141, 168, 205, 207, 216, 217, 218, 228, 230
10	12, 74, 93, 116, 119, 121, 126, 138, 177, 203, 206, 215
11	66, 82, 85, 148, 166, 201, 222, 227
12	51, 81, 89, 112, 115, 129, 134, 136, 162, 164, 188, 190
13	16, 67, 84, 111, 125, 147, 193, 194, 202, 204, 223, 224
14	49, 128, 226
15	10, 69, 71, 132, 140, 187, 189
16	87, 176
17	124, 192, 200, 225, 229
18	65, 127, 131, 139
21	174
22	221
24	83, 191
27	47, 123, 175

d: the rank of the abelian group formed by the set of band structures.

Supplementary Table 7. Symmetry-based indicators of band topology for systems with significant spin-orbit coupling and no time-reversal symmetry.

X_{BS}	Space groups
$\mathbb{Z}_2$	3, 11, 14, 48, 49, 50, 52, 53, 54, 56, 57, 58, 59, 60, 61, 62, 63, 64, 66, 67, 68, 70, 72, 73, 74, 77, 79, 111, 112, 113, 114, 115, 116, 117, 118, 119, 120, 121, 122, 125, 126, 129, 130, 133, 134, 137, 138, 141, 142, 162, 163, 164, 165, 166, 167, 171, 172, 201, 203, 205, 206, 215, 216, 217, 218, 219, 220, 222, 224, 227, 228, 230
$\mathbb{Z}_3$	143, 173, 188, 190
$\mathbb{Z}_4$	69, 71, 75, 124, 128, 132, 135, 136, 140, 202, 204, 223, 226
$\mathbb{Z}_6$	168, 192, 193, 194
$\mathbb{Z}_8$	139, 225, 229
$\mathbb{Z}_2 \times \mathbb{Z}_2$	12, 13, 15, 51, 55, 86, 88
$\mathbb{Z}_2 \times \mathbb{Z}_4$	65, 84, 85, 131, 148, 200
$\mathbb{Z}_2 \times \mathbb{Z}_{12}$	147
$\mathbb{Z}_3 \times \mathbb{Z}_3$	187, 189
$\mathbb{Z}_3 \times \mathbb{Z}_6$	176
$\mathbb{Z}_4 \times \mathbb{Z}_4$	87, 127
$\mathbb{Z}_4 \times \mathbb{Z}_8$	221
$\mathbb{Z}_6 \times \mathbb{Z}_{12}$	191
$\mathbb{Z}_2 \times \mathbb{Z}_2 \times \mathbb{Z}_2$	10, 82
$\mathbb{Z}_2 \times \mathbb{Z}_2 \times \mathbb{Z}_4$	81
$\mathbb{Z}_2 \times \mathbb{Z}_4 \times \mathbb{Z}_8$	123
$\mathbb{Z}_3 \times \mathbb{Z}_3 \times \mathbb{Z}_3$	174
$\mathbb{Z}_4 \times \mathbb{Z}_4 \times \mathbb{Z}_4$	83
$\mathbb{Z}_6 \times \mathbb{Z}_6 \times \mathbb{Z}_6$	175
$\mathbb{Z}_2 \times \mathbb{Z}_2 \times \mathbb{Z}_2 \times \mathbb{Z}_4$	2, 47

X_{BS}: the quotient group between the group of band structures and atomic insulators.

Supplementary Table 8. Symmetry-based indicators of band topology for systems of spinless fermions without time-reversal symmetry.

X_{BS}	Space groups
$\mathbb{Z}_2$	3, 11, 14, 27, 37, 45, 48, 49, 50, 52, 53, 54, 56, 58, 60, 61, 66, 68, 70, 73, 77, 79, 103, 104, 106, 110, 112, 114, 116, 117, 118, 120, 122, 126, 130, 133, 142, 162, 163, 164, 165, 166, 167, 171, 172, 184, 201, 203, 205, 206, 218, 219, 220, 222, 228, 230
$\mathbb{Z}_3$	143, 173, 188, 190
$\mathbb{Z}_4$	75, 124, 128
$\mathbb{Z}_6$	168, 192
$\mathbb{Z}_2 \times \mathbb{Z}_2$	12, 13, 15, 86, 88
$\mathbb{Z}_2 \times \mathbb{Z}_4$	84, 85, 148
$\mathbb{Z}_2 \times \mathbb{Z}_{12}$	147
$\mathbb{Z}_3 \times \mathbb{Z}_6$	176
$\mathbb{Z}_4 \times \mathbb{Z}_4$	87
$\mathbb{Z}_2 \times \mathbb{Z}_2 \times \mathbb{Z}_2$	10, 82
$\mathbb{Z}_2 \times \mathbb{Z}_2 \times \mathbb{Z}_4$	81
$\mathbb{Z}_3 \times \mathbb{Z}_3 \times \mathbb{Z}_3$	174
$\mathbb{Z}_4 \times \mathbb{Z}_4 \times \mathbb{Z}_4$	83
$\mathbb{Z}_6 \times \mathbb{Z}_6 \times \mathbb{Z}_6$	175
$\mathbb{Z}_2 \times \mathbb{Z}_2 \times \mathbb{Z}_2 \times \mathbb{Z}_4$	2

X_{BS}: the quotient group between the group of band structures and atomic insulators.

Supplementary Table 9. **Characterization of band structures for quasi-two-dimensional systems with significant spin-orbit coupling and time-reversal symmetry.**

d	Layer groups
1	1, 3, 4, 5, 8, 9, 10, 11, 12, 13, 19, 20, 21, 22, 23, 24, 25, 26, 27, 28, 29, 30, 31, 32, 33, 34 35, 36
2	39, 43, 45, 46, 54, 56, 58, 60
3	7, 15, 16, 17, 38, 40, 41, 42, 44, 48, 49, 50, 53, 55, 57, 59, 68, 70
4	18, 47, 52, 62, 64, 65, 67, 69, 73, 76, 77
5	2, 6, 14, 37, 63, 79
6	66, 71, 72
7	74, 78
8	51, 61
9	75, 80

d: the rank of the abelian group formed by the set of band structures.

Supplementary Table 10. **Characterization of band structures for quasi-two-dimensional systems with negligible spin-orbit coupling and time-reversal symmetry.**

d	Layer groups
1	1, 5, 9, 12, 33
2	4, 10, 13, 29, 32, 34
3	8, 11, 17, 21, 25, 28, 30, 31, 36
4	15, 16, 20, 24, 35, 43, 45, 65, 68, 70
5	2, 3, 7, 54, 56, 58, 60, 67, 69
6	18, 22, 26, 27, 39, 42, 44, 46, 49, 50, 52, 66, 73
8	40, 71, 72, 74, 76, 77, 79
9	14, 19, 23, 38, 41, 48, 53, 55, 57, 59, 62, 64
10	6, 63, 78
12	47, 51, 75
16	80
18	37, 61

d: the rank of the abelian group formed by the set of band structures.

Supplementary Table 11. **Characterization of band structures for quasi-two-dimensional systems with significant spin-orbit coupling and no time-reversal symmetry.**

d	Layer groups
1	1, 5, 9, 12, 19, 23, 27, 28, 29, 30, 33, 35, 36
2	4, 10, 13, 22, 26, 32, 34, 39, 46
3	8, 11, 17, 21, 25, 31, 43, 45, 48, 53, 55, 57, 59
4	15, 16, 20, 24, 38, 41, 54, 56, 58, 60, 62, 64, 76, 77
5	2, 3, 7, 37, 40, 44, 47, 67, 68, 69, 70
6	18, 42
7	65, 78, 79
8	49, 50, 52, 61, 63, 71, 72
9	14, 66, 73, 80
10	6
14	74
16	51
18	75

d: the rank of the abelian group formed by the set of band structures.

Supplementary Table 12. Characterization of band structures for quasi-two-dimensional systems of spinless femrions without time-reversal symmetry.

d	Layer groups
1	1, 5, 9, 12, 33
2	4, 10, 13, 29, 32, 34
3	8, 11, 17, 21, 25, 28, 30, 31, 36
4	15, 16, 20, 24, 35, 43, 45
5	2, 3, 7, 67, 68, 69, 70
6	18, 22, 26, 27, 39, 42, 44, 46, 54, 56, 58, 60
7	65
8	40, 49, 50, 52, 71, 72, 76, 77
9	14, 19, 23, 38, 41, 48, 53, 55, 57, 59, 62, 64, 66, 73
10	6, 78, 79
12	47, 63
14	74
16	51, 80
18	37, 61, 75

d: the rank of the abelian group formed by the set of band structures.

Supplementary Table 13. Characterization of band structures for quasi-one-dimensional systems with significant spin-orbit coupling and time-reversal symmetry.

d	Rod groups
1	1, 3, 4, 5, 8, 9, 10, 13, 14, 15, 16, 17, 18, 19, 24, 25, 26, 31, 32, 33, 35, 43, 44, 47, 48, 54<br>55, 57, 58, 63, 64, 66, 67
2	7, 12, 21, 22, 23, 30, 34, 36, 38, 42, 46, 49, 50, 56, 65, 70
3	2, 6, 11, 20, 27, 29, 37, 41, 53, 62, 68, 69, 72
4	40, 52, 59, 71
5	61, 75
6	28, 39, 45, 51, 74
9	60, 73

d: the rank of the abelian group formed by the set of band structures.

Supplementary Table 14. Characterization of band structures for quasi-one-dimensional systems with negligible spin-orbit coupling and time-reversal symmetry.

d	Rod groups
1	1, 5, 9, 24, 26, 43, 44, 54, 58
2	4, 8, 16, 17, 25, 42, 50, 55, 56, 57
3	2, 3, 7, 10, 12, 14, 19, 23, 31, 33, 35, 36, 47, 48, 49, 63, 67, 70
4	15, 27, 46, 53, 65, 69
5	29, 34, 38, 52, 72
6	6, 11, 13, 18, 21, 22, 32, 45, 59, 61, 64, 66, 68
7	30, 37
8	40, 62
9	28, 41, 51, 71, 75
10	74
12	20, 60
15	39
18	73

d: the rank of the abelian group formed by the set of band structures.

Supplementary Table 15. Characterization of band structures for quasi-one-dimensional systems with significant spin-orbit coupling and no time-reversal symmetry.

d	Rod groups
1	1, 5, 9, 13, 15, 16, 17, 18, 24, 26, 32, 35, 43, 44, 54, 58, 64, 66
2	4, 8, 25, 30, 34, 36, 50, 55, 57, 70
3	2, 3, 7, 10, 12, 14, 19, 20, 21, 22, 31, 33, 37, 38, 41, 42, 47, 48, 49, 56, 62, 63, 67, 68, 69
4	23, 46, 65, 71
6	6, 11, 27, 29, 39, 40, 52, 53, 72, 75
9	45, 51, 59, 61, 73, 74
12	28
18	60

d: the rank of the abelian group formed by the set of band structures.

Supplementary Table 16. Characterization of band structures for quasi-one-dimensional systems of spinless fermions without time-reversal symmetry.

d	Rod groups
1	1, 5, 9, 24, 26, 43, 44, 54, 58
2	4, 8, 16, 17, 25, 50, 55, 57
3	2, 3, 7, 10, 12, 14, 19, 31, 33, 35, 36, 42, 47, 48, 49, 56, 63, 67, 70
4	15, 23, 46, 65, 69
5	34
6	6, 11, 13, 18, 21, 22, 27, 29, 32, 38, 52, 53, 64, 66, 68, 72
7	30, 37
8	62
9	40, 41, 45, 51, 59, 61, 71, 75
12	20, 28, 74
15	39
18	60, 73

d: the rank of the abelian group formed by the set of band structures.

Supplementary Table 17. Symmetry-based indicators of band topology for quasi-two-dimensional systems with significant spin-orbit coupling and time-reversal symmetry.

X_{BS}	Layer groups
$\mathbb{Z}_2$	2, 6, 7, 14, 15, 16, 17, 18, 37, 38, 39, 40, 41, 42, 43, 44, 45, 46, 47, 48, 52, 62, 64, 66, 71, 72
$\mathbb{Z}_3$	74, 78, 79
$\mathbb{Z}_4$	51, 61, 63
$\mathbb{Z}_6$	75, 80

X_{BS}: the quotient group between the group of band structures and atomic insulators.

Supplementary Table 18. Symmetry-based indicators of band topology for quasi-two-dimensional systems with negligible spin-orbit coupling and time-reversal symmetry.

X_{BS}	Layer groups
$\mathbb{Z}_2$	2, 3, 7, 49, 50, 52, 66, 73
$\mathbb{Z}_2 \times \mathbb{Z}_2$	6, 51, 75

X_{BS}: the quotient group between the group of band structures and atomic insulators.

Supplementary Table 19. Symmetry-based indicators of band topology for quasi-two-dimensional systems with significant spin-orbit coupling and no time-reversal symmetry.

$X_{\rm BS}$	Layer groups
$\mathbb{Z}_2$	2, 3, 7, 37, 40, 44, 47
$\mathbb{Z}_3$	65, 78, 79
$\mathbb{Z}_4$	49, 50, 52, 61, 63
$\mathbb{Z}_6$	66, 73, 80
$\mathbb{Z}_2 \times \mathbb{Z}_2$	6
$\mathbb{Z}_3 \times \mathbb{Z}_3$	74
$\mathbb{Z}_4 \times \mathbb{Z}_4$	51
$\mathbb{Z}_6 \times \mathbb{Z}_6$	75

$X_{\rm BS}$: the quotient group between the group of band structures and atomic insulators.

Supplementary Table 20. Symmetry-based indicators of band topology for quasi-two-dimensional systems of spinless fermions without time-reversal symmetry.

$X_{\rm BS}$	Layer groups
$\mathbb{Z}_2$	2, 3, 7
$\mathbb{Z}_3$	65
$\mathbb{Z}_4$	49, 50, 52
$\mathbb{Z}_6$	66, 73
$\mathbb{Z}_2 \times \mathbb{Z}_2$	6
$\mathbb{Z}_3 \times \mathbb{Z}_3$	74
$\mathbb{Z}_4 \times \mathbb{Z}_4$	51
$\mathbb{Z}_6 \times \mathbb{Z}_6$	75

$X_{\rm BS}$: the quotient group between the group of band structures and atomic insulators.

Supplementary References

[1] Bradley, C. J. & Cracknell, A. P. *The mathematical theory of symmetry in solids: representation theory for point groups and space groups.* (Oxford University Press, 1972).

[2] Hahn, T. (ed.) *International Tables for Crystallography.* Vol. A: Space-group symmetry, 5th ed. (Springer, 2006).

[3] Michel, L. & Zak, J. Connectivity of energy bands in crystals. *Phys. Rev. B* **59,** 5998–6001 (1999).

[4] Michel, L. & Zak, J. Elementary energy bands in crystalline solids. *Euro Phys. Lett.* **50,** 519–525 (2000).

[5] Michel, L. & Zak, J. Elementary energy bands in crystals are connected. *Phys. Rep.* **341,** 377–395 (2001).

[6] Wieder, B. J. & Kane, C. L. Spin-orbit semimetals in the layer groups. *Phys. Rev. B* **94,** 155108 (2016).

[7] Turner, A. M., Zhang, Y., Mong, R. S. K. & Vishwanath, A., Quantized response and topology of magnetic insulators with inversion symmetry. *Phys. Rev. B* **85,** 165120 (2012).

[8] Hughes, T. L., Prodan, E. & Bernevig, B. A. Inversion-symmetric topological insulators. *Phys. Rev. B* **83,** 245132 (2011).

[9] Freed, D. S. & Moore, G. W. Twisted equivariant matter. *Annales Henri Poincaré* **14,** 1927–2023 (2013).

[10] Kruthoff, J., de Boer, J., van Wezel, J., Kane, C. L. & Slager, R. J. Topological classification of crystalline insulators through band structure combinatorics. *arXiv:1612.02007.*

[11] Watanabe, H., Po, H. C., Vishwanath, A. & Zaletel, M. P., Filling constraints for spin-orbit coupled insulators in symmorphic and nonsymmorphic crystals. *Proc. Natl. Acad. Sci. U.S.A.* **112,** 14551 (2015).

[12] Watanabe, H., Po HC, Zaletel MP & Vishwanath A Filling-enforced gaplessness in band structures of the 230 space groups. *Phys. Rev. Lett.* **117,** 096404 (2016).

[13] Fang, C., Gilbert, M. J. & Bernevig, B. A. Bulk topological invariants in noninteracting point group symmetric insulators. *Phys. Rev. B* **86,** 115112 (2012).

[14] Po, H. C., Watanabe, H., Zaletel, M. P. & Vishwanath, A. Filling-enforced quantum band insulators in spin-orbit coupled crystals. *Sci. Adv.* **2,** e1501782 (2016).
